# A Comprehensive Review of Cardiovascular Disease Management: Cardiac Biomarkers, Imaging Modalities, Pharmacotherapy, Surgical Interventions, and Herbal Remedies

**DOI:** 10.3390/cells13171471

**Published:** 2024-09-01

**Authors:** Vasudeva Reddy Netala, Sireesh Kumar Teertam, Huizhen Li, Zhijun Zhang

**Affiliations:** 1 School of Chemical Engineering and Technology, North University of China, Taiyuan 030051, Chinahzli@nuc.edu.cn (H.L.); 2 Department of Dermatology, School of Medicine and Public Health, University of Wisconsin-Madison, Madison, WI 53705, USA

**Keywords:** cardiac biomarkers, imaging modalities, MRI, ACE inhibitors, herbal remedies, flavonoids, phenolic acids, saponins, cardioprotective effects

## Abstract

Cardiovascular diseases (CVDs) continue to be a major global health concern, representing a leading cause of morbidity and mortality. This review provides a comprehensive examination of CVDs, encompassing their pathophysiology, diagnostic biomarkers, advanced imaging techniques, pharmacological treatments, surgical interventions, and the emerging role of herbal remedies. The review covers various cardiovascular conditions such as coronary artery disease, atherosclerosis, peripheral artery disease, deep vein thrombosis, pulmonary embolism, cardiomyopathy, rheumatic heart disease, hypertension, ischemic heart disease, heart failure, cerebrovascular diseases, and congenital heart defects. The review presents a wide range of cardiac biomarkers such as troponins, C-reactive protein, CKMB, BNP, NT-proBNP, galectin, adiponectin, IL-6, TNF-α, miRNAs, and oxylipins. Advanced molecular imaging techniques, including chest X-ray, ECG, ultrasound, CT, SPECT, PET, and MRI, have significantly enhanced our ability to visualize myocardial perfusion, plaque characterization, and cardiac function. Various synthetic drugs including statins, ACE inhibitors, ARBs, β-blockers, calcium channel blockers, antihypertensives, anticoagulants, and antiarrhythmics are fundamental in managing CVDs. Nonetheless, their side effects such as hepatic dysfunction, renal impairment, and bleeding risks necessitate careful monitoring and personalized treatment strategies. In addition to conventional therapies, herbal remedies have garnered attention for their potential cardiovascular benefits. Plant extracts and their bioactive compounds, such as flavonoids, phenolic acids, saponins, and alkaloids, offer promising cardioprotective effects and enhanced cardiovascular health. This review underscores the value of combining traditional and modern therapeutic approaches to improve cardiovascular outcomes. This review serves as a vital resource for researchers by integrating a broad spectrum of information on CVDs, diagnostic tools, imaging techniques, pharmacological treatments and their side effects, and the potential of herbal remedies.

## 1. Introduction

Cardiovascular diseases (CVDs) refer to a group of disorders affecting the heart and blood vessels, including conditions such as coronary artery disease, stroke, hypertension, and peripheral artery disease. These conditions lead to poor quality of life and severe health complications, such as heart attacks, strokes, heart failure, and arrhythmias, and often result in long-term disability [1,2,3,4]. The impact of CVD extends beyond physical health, affecting emotional well-being and imposing significant economic burdens on individuals, families, and healthcare systems worldwide. Protecting oneself from these debilitating diseases requires a multifaceted approach, including maintaining a healthy lifestyle and managing risk factors [5,6]. Adopting a balanced diet rich in fruits, vegetables, whole grains, and lean proteins while limiting saturated fats, trans fats, and sodium is crucial. Regular physical activity and moderate-intensity exercise helps keep the heart and blood vessels healthy. Avoiding tobacco use and limiting alcohol consumption are also vital steps in preventing CVDs. Additionally, managing stress through relaxation techniques, adequate sleep, and social support plays a significant role in heart health. For individuals with risk factors, such as high blood pressure, high cholesterol, or diabetes, regular monitoring and appropriate medical management are essential. Routine check-ups with healthcare providers can help detect early signs of cardiovascular issues and enable timely intervention [6,7,8,9].

However, the prevalence of CVDs among elderly populations in low- and middle-income countries (LMICs) has surged, posing a formidable challenge to the sustainability of healthcare systems. According to the WHO, CVDs account for 17.9 million deaths annually, representing 32% of all global deaths. Of these deaths, 85% were due to heart attack and stroke. This alarming trend is exacerbated by several factors unique to LMICs, including limited access to quality healthcare, insufficient public health infrastructure, and higher prevalence of risk factors such as hypertension, diabetes, and smoking [1,2,3,4,5,6,7,8,9,10]. Furthermore, the socioeconomic burden of CVD is profound, leading to increased healthcare costs, loss of productivity, and financial strain on families and communities. As populations in LMICs continue to age, the need for effective prevention, early detection, and management of CVDs becomes ever more critical. Integrating CVD care into primary healthcare, improving health education, and implementing policies to reduce risk factors are essential strategies to mitigate the growing impact of cardiovascular diseases in these regions. Addressing these challenges requires coordinated efforts from governments, healthcare providers, and international organizations to ensure that all individuals, regardless of socioeconomic status, have access to the care and resources necessary to prevent and manage CVDs effectively. 

The present review encompasses a wide spectrum of conditions such as coronary artery disease, atherosclerosis, peripheral artery disease, deep vein thrombosis, pulmonary embolism, cardiomyopathy, rheumatic heart disease, hypertension, ischemic heart disease, myocardial infarction, heart failure, cerebrovascular diseases, and congenital heart defects. It highlights an array of cardiac biomarkers, including troponins, C-reactive protein, CKMB, BNP, NT-proBNP, galectin, adiponectin, IL-6, TNF-α, miRNAs, oxylipins, and LDL/HDL, which are pivotal in the early diagnosis, risk stratification, and management of these conditions. In parallel, advanced molecular imaging techniques like chest X-ray, ECG, ultrasound, CT, SPECT, PET, and MRI have significantly enhanced our ability to visualize myocardial perfusion, plaque characterization, and cardiac function, offering critical insights for personalized treatment strategies. The role of synthetic drugs, such as statins, ACE inhibitors, ARBs, β-blockers, calcium channel blockers, antihypertensives, anticoagulants, and antiarrhythmics, remains fundamental in managing CVDs. However, their associated side effects, including hepatic dysfunction, renal impairment, and bleeding risks, highlight the necessity for careful monitoring and individualized treatment plans. Amidst these conventional therapies, herbal remedies have garnered increasing attention for their potential cardiovascular benefits. Bioactive compounds from plant extracts, such as flavonoids, phenolic acids, saponins, and alkaloids, have shown promising cardioprotective effects, offering a complementary approach to enhance cardiovascular health. This study aims to bridge this gap by exploring the synergistic use of novel biomarkers and advanced imaging modalities alongside traditional and emerging therapeutic approaches, offering a distinctive perspective on the management of CVDs. This review underscores the importance of integrating traditional and modern therapeutic approaches to improve cardiovascular outcomes, addressing existing gaps in the literature and paving the way for more effective and personalized treatment strategies. Despite the vast amount of research on CVDs, there remains a critical need for a holistic perspective that bridges traditional and modern therapeutic approaches, especially in the context of novel biomarkers and advanced imaging modalities. This review aims to fill this gap by offering an in-depth analysis of the current state of knowledge, identifying existing challenges, and proposing new directions for research and clinical practice. This study advances knowledge by providing a unique synthesis of information that combines traditional cardiovascular treatments with emerging strategies, including the use of herbal remedies and bioactive compounds. Additionally, it emphasizes the role of cutting-edge imaging technologies and novel biomarkers in enhancing early diagnosis, risk stratification, and personalized treatment. By addressing these elements comprehensively, this review not only consolidates existing knowledge but also paves the way for more effective and integrated approaches to managing CVDs.

## 2. Diverse Manifestations of Cardiovascular Diseases

CVDs encompass a broad range of conditions that affect the heart and blood vessels, posing significant health risks and leading to substantial morbidity and mortality worldwide. These diseases can arise from a variety of mechanisms, including structural abnormalities, blockages, such as coronary artery stenosis and occlusion, and dysfunctions within the cardiovascular system, affecting the heart’s blood vessels, endocardium, myocardium, and pericardium. Understanding the intricate mechanisms and health impacts of these conditions is crucial for effective prevention, diagnosis, and treatment. An in-depth look at several major cardiovascular diseases, detailing their underlying mechanisms and their effects on health, was presented.

### 2.1. Coronary Artery Disease (CAD)

**Figure 1:**
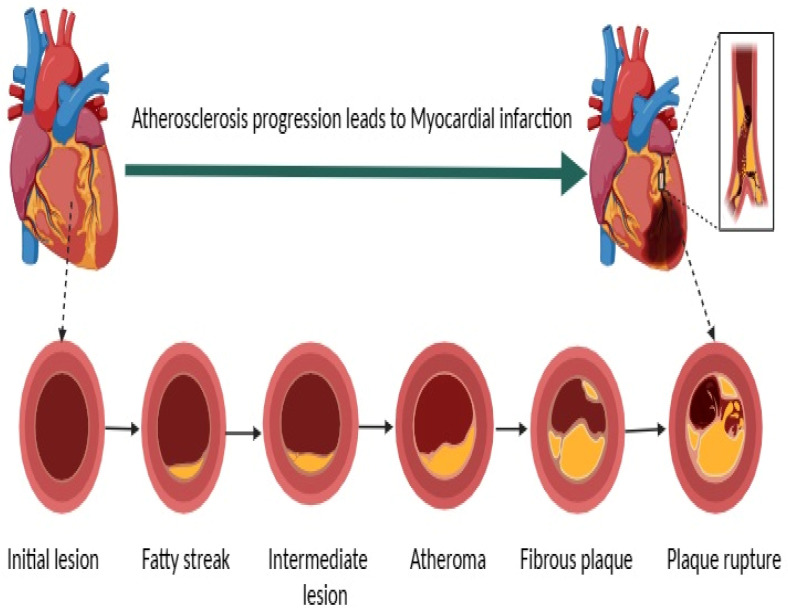
The progression of atherosclerosis through various stages that lead to MI (heart attack); the various stages include initial lesion, fatty streak, intermediated lesion, atheroma, fibrous plaque, and plaque rupture. (Figure is created using app.biorender.com, accessed on 15 May 2024).

CAD is primarily caused by atherosclerosis, a condition where plaque composed of fat, cholesterol, and other substances builds up on the walls of the arteries. Atherosclerosis is the process of plaque buildup within the arterial walls throughout the body. This chronic inflammatory condition begins with endothelial injury, leading to lipid accumulation, immune cell infiltration, and smooth muscle cell proliferation. Over time, the plaque hardens and narrows the arteries, restricting blood flow. Plaques can also become unstable and rupture, causing thrombosis and acute events like MI and stroke. Atherosclerosis is a major underlying cause of various CVDs, contributing to morbidity and mortality through complications such as heart attack, stroke, and peripheral artery disease [11,12,13,14]. The progression of atherosclerosis (Figure 1), the underlying cause of CAD, unfolds through several distinct stages. Initially, the lesion is characterized by normal histology with the infiltration of macrophages. This is followed by the fatty streak stage, marked by intracellular lipid accumulation. As the disease progresses, the intermediate lesion stage sees both intracellular and extracellular lipid accumulation. The development of an extracellular lipid core signifies the atheroma stage. Subsequently, fibrotic and calcific layers form over the atheroma, contributing to arterial narrowing in the fibrous plaque stage. The final stage involves plaque rupture, thrombosis, and blockage of blood flow, leading to myocardial infarction (heart attack), and it is known as the complicated lesion or rupture stage.

### 2.2. Peripheral Arterial Disease (PAD)

**Figure 2:**
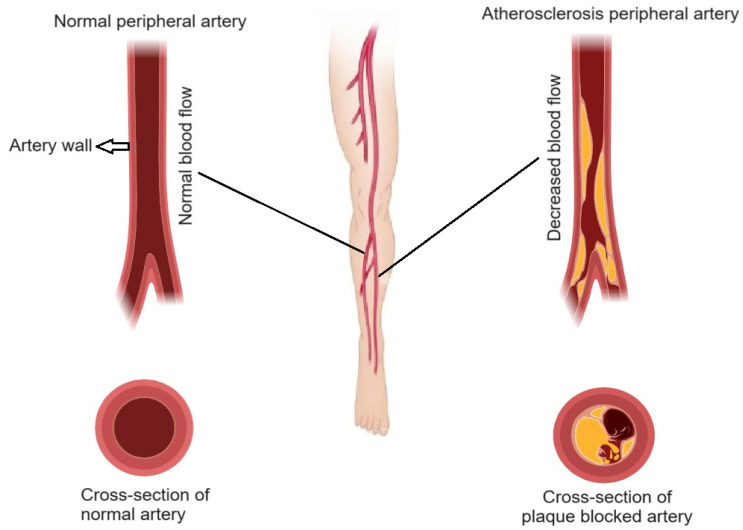
A comparison between a leg with a normal, healthy peripheral artery showing normal blood flow and with an atherosclerotic peripheral artery exhibiting decreased blood flow due to a plaque-blocked artery. (Figure is created using app.biorender.com, accessed on 15 May 2024).

PAD occurs when atherosclerosis affects the arteries that supply blood to the limbs, usually the legs (Figure 2). The narrowed arteries reduce blood flow, leading to symptoms such as leg pain while walking (claudication), numbness, and weakness. Severe PAD can cause critical limb ischemia, characterized by pain at rest, non-healing wounds, and tissue death (gangrene), potentially necessitating amputation. PAD is a significant risk factor for cardiovascular events, including heart attacks and strokes, and can substantially impair quality of life and mobility [15]. Early detection and management of PAD are crucial to prevent progression and complications. Treatment options include lifestyle changes, medication, and in severe cases, revascularization procedures to restore blood flow. Regular monitoring and comprehensive cardiovascular risk management are essential for improving outcomes in PAD patients [16].

### 2.3. Deep Vein Thrombosis (DVT) and Pulmonary Embolism (PE)

**Figure 3:**
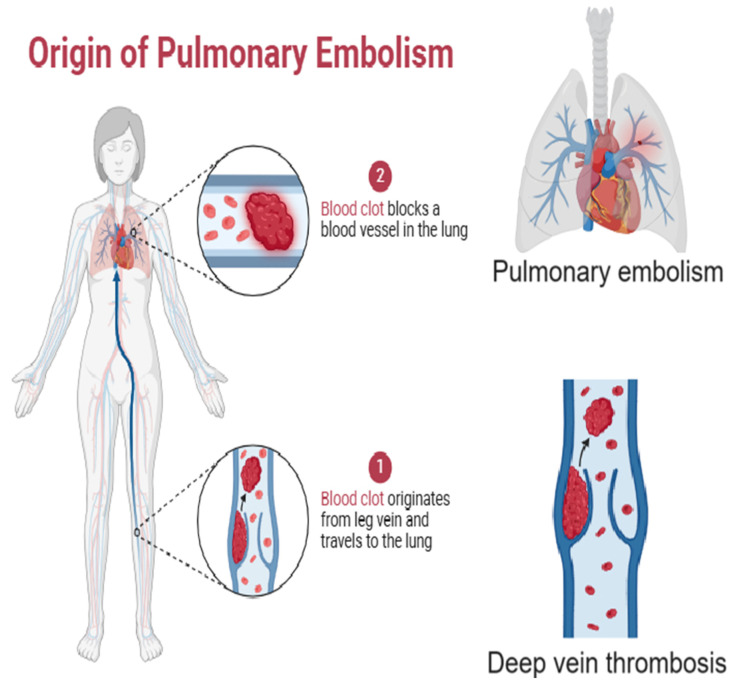
This figure clearly illustrates how deep vein thrombosis (DVT) can lead to a pulmonary embolism (PE). A blood clot originates in a leg vein (DVT) and travels to the lung, where it blocks a blood vessel, resulting in a serious cardiovascular condition known as PE. (Figure is created using app.biorender.com, accessed on 15 May 2024).

DVT involves the formation of a blood clot in a deep vein, typically in the legs. The clot can block blood flow, causing pain, swelling, redness, and warmth in the affected limb. If a part of the clot breaks off, it can travel through the bloodstream and lodge in the lungs, causing PE, a life-threatening condition (Figure 3). DVT and PE together constitute venous thromboembolism (VTE), which can lead to long-term complications, such as post-thrombotic syndrome (chronic pain and swelling) and pulmonary hypertension, significantly affecting a person’s health and mobility [17]. PE occurs when a blood clot, usually originating from DVT, travels to the lungs and blocks a pulmonary artery. This blockage can severely impair oxygen exchange and blood flow, leading to symptoms like sudden shortness of breath, chest pain, rapid heart rate, and coughing up blood. Severe PE can cause right ventricular failure and death if not promptly treated. Chronic complications include pulmonary hypertension, which leads to persistent shortness of breath and exercise intolerance. PE requires immediate medical attention and can have lasting impacts on respiratory and cardiovascular health [18].

### 2.4. Cardiomyopathies

**Figure 4:**
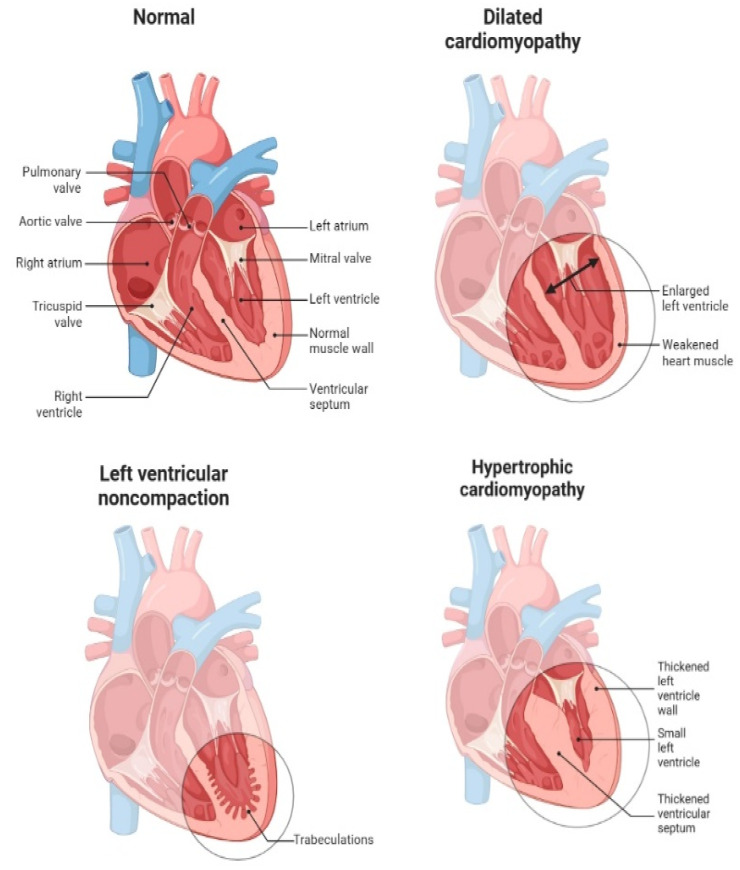
Representation of different types of cardiomyopathies, including dilated cardiomyopathy, left ventricular noncompaction, and hypertrophic cardiomyopathy. Figure is created using app.biorender.com, accessed on 15 May 2024.

Cardiomyopathies are diseases of the heart muscle that affect its size, shape, structure, and function. They can be inherited or acquired and include dilated, hypertrophic, and restrictive forms. These conditions lead to impaired cardiac function, resulting in HF, arrhythmias, and sudden cardiac death. Symptoms often include fatigue, shortness of breath, swelling of the legs and ankles, and palpitations [19]. Cardiomyopathies can severely affect a person’s ability to perform daily activities and require long-term management with medications, lifestyle changes, and potentially device implantation or heart transplantation. Early diagnosis and treatment plans are crucial for improving prognosis and quality of life in patients with cardiomyopathies. Genetic counseling and screening of family members may be recommended to identify those at risk and initiate preventive measures [20]. Different cardiomyopathies include dilated cardiomyopathy (DCM), characterized by an enlarged and weakened heart, reducing its ability to pump blood efficiently. It often leads to heart failure and arrhythmias. For hypertrophic cardiomyopathy (HCM), particularly the obstructive form (H(O)CM), the outflow tract of the left ventricle can become obstructed due to the thickening of the heart muscle, specifically the interventricular septum. This obstruction can lead to increased pressure within the heart, reduced cardiac output, and symptoms such as chest pain, dyspnea, and syncope. Restrictive Cardiomyopathy (RCM) occurs when the heart muscle becomes rigid and less elastic, preventing proper filling of the heart chambers. This condition often leads to heart failure and arrhythmias. Arrhythmogenic Right Ventricular Cardiomyopathy (ARVC) is a rare type where the muscle of the right ventricle is replaced by fat and fibrous tissue, leading to arrhythmias and an increased risk of sudden cardiac death. Left ventricular noncompaction cardiomyopathy (LVNC) is characterized by the presence of trabeculations in the left ventricle, leading to impaired heart function. It can cause heart failure, arrhythmias, and thromboembolic events. Takotsubo Cardiomyopathy, also known as stress-induced cardiomyopathy or “broken heart syndrome”, is typically triggered by severe emotional or physical stress. It causes temporary weakening of the heart muscle, usually the left ventricle [19,20]. Figure 4 represents different types of dilated cardiomyopathies, hypertrophics, and left ventricular noncompaction cardiomyopathy. 

### 2.5. Cerebrovascular Diseases

**Figure 5:**
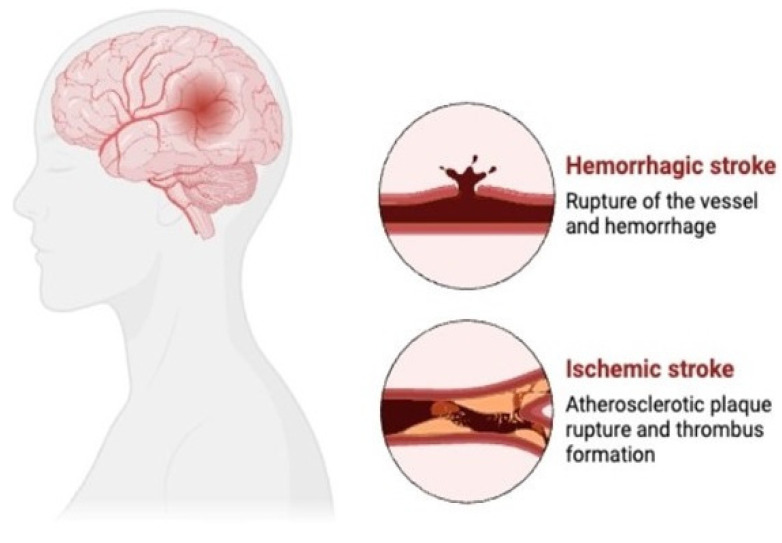
Different types of strokes, such as atherosclerosis strokes; atherosclerotic plaque and artery blockages; hemorrhagic strokes; ruptures of the vessel; and hemorrhages. (Figure is created using app.biorender.com, accessed on 15 May 2024).

Stroke occurs when the blood supply to a part of the brain is interrupted or reduced, depriving brain tissue of oxygen and nutrients. This can be due to a blocked artery (ischemic stroke), the rupture of a blood vessel (hemorrhagic stroke), or atherosclerotic plaque buildup and artery blockage (atherosclerosis stroke/ischemic stroke), which are represented in Figure 5. Rapid intervention is critical to minimize brain damage and improve outcomes. Symptoms include sudden weakness or numbness, confusion, difficulty speaking, vision problems, and severe headache. Long-term effects include physical and cognitive impairments, emotional disturbances, and an increased risk of recurrent strokes, profoundly impacting independence and quality of life [21].

### 2.6. Rheumatic Heart Disease (RHD)

**Figure 6:**
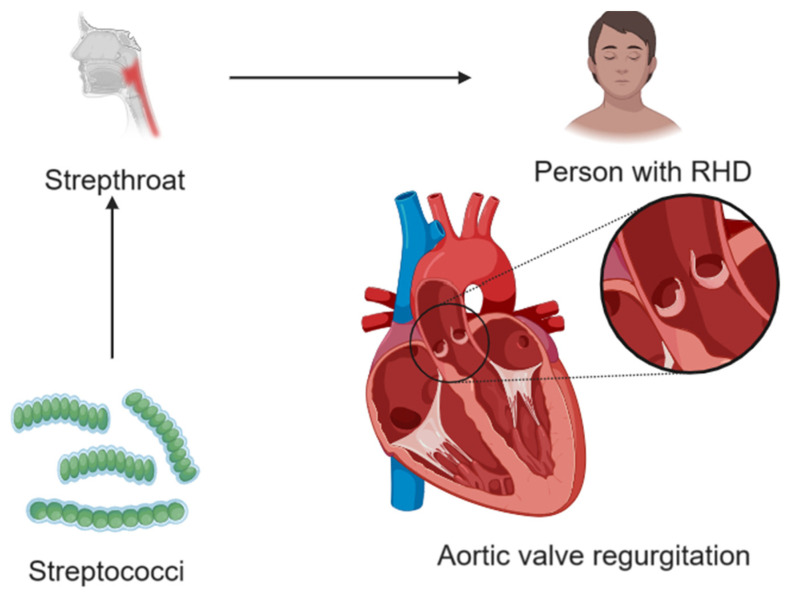
A depiction of rheumatic heart disease (RHD), illustrating the sequence from streptococcal infection and strep throat to an individual with RHD and aortic valve regurgitation. (Figure is created using app.biorender.com, accessed on 15 June 2024).

RHD is a chronic condition resulting from acute rheumatic fever, an inflammatory disease triggered by a streptococcal infection, typically in childhood. The disease primarily affects the heart valves, leading to scarring and deformation, which can cause valve stenosis or regurgitation (Figure 6). Symptoms of RHD can include shortness of breath, chest pain, fatigue, and swelling in the legs and abdomen due to HF. If left untreated, RHD can lead to severe complications such as atrial fibrillation, infective endocarditis, and stroke. Management of RHD often involves long-term use of antibiotics to prevent recurrent rheumatic fever, medications to manage HF and arrhythmias, and surgical interventions, such as valve repair or replacement, in advanced cases. Preventing rheumatic fever through early treatment of streptococcal throat infections is crucial in reducing the incidence of rheumatic heart disease, especially in regions where the disease remains endemic [22,23].

### 2.7. Congenital Heart Defects (CHDs)

CHDs are structural abnormalities of the heart present at birth, resulting from improper development during fetal life. These defects can range from simple issues, such as small holes between heart chambers (atrial or ventricular septal defects), to complex malformations, involving multiple structures and abnormal blood flow patterns [24]. Common types of CHDs include atrial septal defect (ASD), ventricular septal defect (VSD), patent ductus arteriosus (PDA), and Tetralogy of Fallot (TOF) [25]. ASD and VSD involve abnormal openings in the heart’s septum, allowing oxygen-rich and oxygen-poor blood to mix, which can strain the heart and lungs. PDA is a condition where the ductus arteriosus, a blood vessel that bypasses the lungs in fetal circulation, fails to close after birth, leading to excessive blood flow to the lungs and heart overwork [26]. TOF is a more complex defect consisting of four abnormalities: a VSD, pulmonary stenosis (narrowing of the pulmonary valve), right ventricular hypertrophy (thickening of the right ventricle), and an overriding aorta (aorta positioned above the VSD). This combination results in insufficient oxygenated blood reaching the body, causing cyanosis [27]. Other notable CHDs include coarctation of the aorta (narrowing of the aorta), transposition of the great arteries (TGA), where the positions of the pulmonary artery and the aorta are switched, and hypoplastic left heart syndrome (HLHS), where the left side of the heart is underdeveloped [28]. Each type of CHD can lead to a variety of health issues, including cyanosis (bluish skin due to low oxygen levels), HF, arrhythmias, and impaired growth and development in children. Treatment often requires surgical intervention, such as repairing or closing septal defects, widening narrowed blood vessels, or reconstructing heart chambers. Long-term medical care, including regular monitoring, medications, and lifestyle adjustments, is essential to manage symptoms and prevent complications [24,25,26,27,28,29].

### 2.8. Myocardial Infarction (MI)

MI, commonly known as a heart attack, occurs when blood flow to a part of the heart muscle is blocked, usually by a blood clot forming on a ruptured atherosclerotic plaque in a coronary artery. The lack of oxygenated blood leads to the death of heart muscle tissue. Symptoms include severe chest pain, often radiating to the arm, shoulder, back, or jaw, shortness of breath, sweating, nausea, and light headedness [30,31]. In some cases, symptoms can be atypical, especially in women, elderly individuals, and those with diabetes. Immediate medical intervention is crucial to restore blood flow and minimize heart damage, typically involving the use of various medications, like thrombolytics, antiplatelet agents, and anticoagulants, as well as procedures, such as angioplasty and stent placement or coronary artery bypass grafting (CABG). Long-term effects can include HF, arrhythmias, and an increased risk of future cardiovascular events, significantly impacting overall health and quality of life. Comprehensive rehabilitation, lifestyle modifications, and adherence to prescribed medications are essential for recovery and prevention of recurrent events [32,33].

### 2.9. Cardiac Arrhythmias

Cardiac arrhythmias are disorders of the heart’s electrical system that result in irregular heartbeats, which can be too fast (tachycardia), too slow (bradycardia), or irregular (fibrillation). These abnormalities can stem from various factors, including structural heart changes, electrolyte imbalances, or ischemic heart disease. Common types of arrhythmias include atrial fibrillation, atrial flutter, ventricular tachycardia, and ventricular fibrillation. Symptoms may range from palpitations, dizziness, and fatigue to more severe presentations such as syncope, chest pain, and sudden cardiac arrest [34,35]. Management of arrhythmias involves various medications and in certain cases, interventional procedures such as catheter ablation, pacemaker insertion, or implantable cardioverter defibrillators (ICDs) may be necessary. Long-term management focuses on controlling arrhythmia, preventing stroke in cases like atrial fibrillation, and maintaining overall cardiovascular health through lifestyle modifications and regular medical follow-up [36,37].

The following table (Table 1) provides a comprehensive overview of key cardiovascular conditions, detailing their primary causes, associated symptoms, potential complications, and recommended management strategies. By bringing together these critical aspects, the table helps in understanding the complex nature of cardiovascular diseases and the need for personalized treatment approaches.

## 3. Diagnosis of CVD Using Cardiac Biomarkers and Imaging Modalities

Diagnosing CVDs involves a combination of clinical evaluations, cardiac biomarkers, and advanced imaging techniques. Cardiac biomarkers are substances released into the blood when the heart is damaged or stressed, providing crucial information about the presence and severity of heart disease. Imaging modalities complement biomarker analysis by offering detailed visualizations of the heart’s structure and function, helping to identify abnormalities such as blockages, structural defects, and tissue damage. Together, these diagnostic tools enable healthcare professionals to accurately diagnose and manage various forms of CVD, improving patient outcomes.

### 3.1. Cardiac Biomarkers

**Figure 7:**
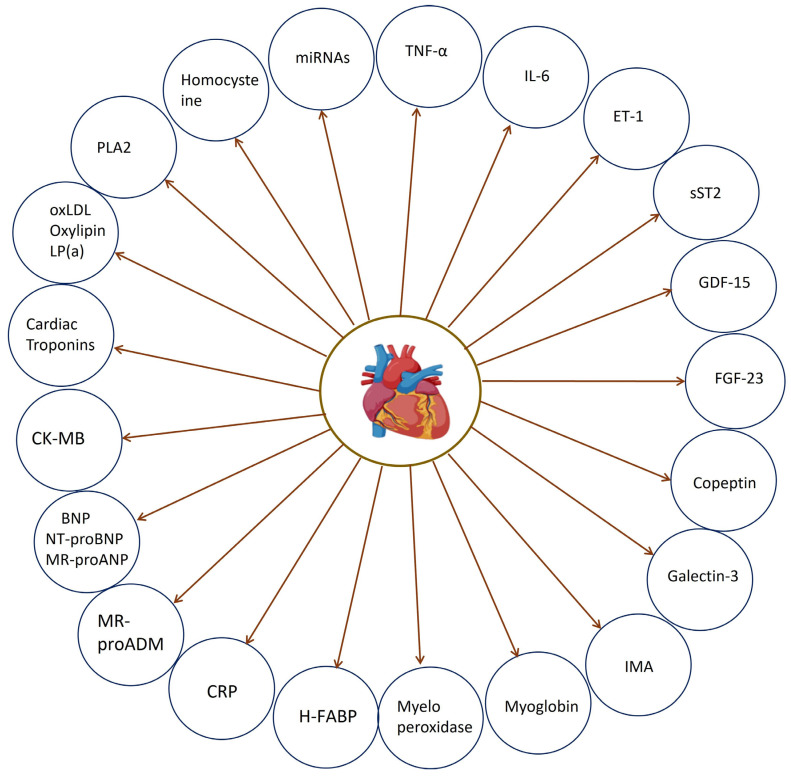
Cardiac biomarkers used in the diagnostic and prognostic evaluation of cardiovascular diseases (CVDs). (Figure is created using app.biorender.com, accessed on 15 June 2024).

The present comprehensive review studied 23 distinct biomarkers, encompassing well-established, pivotal biomarkers, as well as emerging and novel indicators, as clearly depicted in Figure 7. These biomarkers span a wide range of applications, from diagnostic to prognostic and therapeutic uses. This review highlights their potential impact on advancing personalized medicine and improving patient outcomes.

#### 3.1.1. Cardiac Troponins

Cardiac troponins, specifically troponin I (cTnI) and troponin T (cTnT), are proteins released into the bloodstream when the heart muscle is damaged, such as during a heart attack (acute myocardial infarction, AMI). These biomarkers are considered the gold standard for diagnosing AMI due to their high sensitivity and specificity. Elevated troponin levels indicate myocardial injury, and their detection can significantly aid in the early identification and risk stratification of patients presenting with chest pain or other symptoms suggestive of a heart attack. The diagnostic utility of troponins lies in their ability to detect even minor cardiac injuries. Troponin levels start to rise within 3–4 h of myocardial injury and peak at around 24–48 h, and they can remain elevated for up to two weeks. This prolonged elevation allows clinicians to identify recent myocardial damage, which is particularly useful in patients who present late after the onset of symptoms [38,39]. Troponin testing is also valuable in providing prognostic information for patients with chronic heart disease. Persistently elevated troponin levels in these patients can indicate ongoing myocardial stress or injury and may be associated with worse outcomes, such as HF or recurrent cardiac events. Additionally, troponin levels can be used to monitor the effectiveness of therapeutic interventions and guide treatment decisions. Thus, cardiac troponins are crucial biomarkers for diagnosing AMI, offering high sensitivity and specificity. Their prolonged elevation post-injury allows for the identification of recent myocardial damage, and their levels can provide important prognostic information in chronic heart disease, aiding in the comprehensive management of cardiac patients [38,39,40].

#### 3.1.2. Creatine Kinase-MB (CK-MB)

CK-MB is an enzyme primarily found in heart muscle cells, and its presence in the blood is used as a biomarker to indicate myocardial damage, such as that which occurs during a heart attack. CK-MB levels begin to rise within 4–6 h after the onset of myocardial injury, peak at around 18–24 h, and return to baseline within 48–72 h. This rapid rise and fall make CK-MB particularly useful for detecting reinfarction, or a second heart attack occurring shortly after the first. However, CK-MB is less specific than cardiac troponins for diagnosing myocardial injury because it can also be elevated in conditions involving skeletal muscle damage, such as muscular dystrophy, intense physical exercise, trauma, and certain muscle diseases. Despite this, CK-MB remains valuable in conjunction with other tests, especially in scenarios where troponin levels may already be elevated from a prior injury, as it helps in differentiating new cardiac events from ongoing injury [41,42]. Historically, CK-MB has been used alongside other biomarkers to diagnose AMI, and while less specific than troponins, its kinetics—quick rise and fall—are beneficial in acute settings [41,42,43].

#### 3.1.3. B-Type Natriuretic Peptide (BNP)

BNP is a hormone released by the ventricles of the heart in response to excessive stretching of heart muscle cells. High levels of BNP are indicative of HF, and the hormone is particularly useful in differentiating HF from other causes of dyspnea (shortness of breath). BNP provides valuable information in assessing the severity and prognosis of HF [44,45]. Elevated BNP levels are correlated with poor clinical outcomes, and serial measurements of BNP can be used to monitor the effectiveness of treatment and guide therapeutic adjustments. The utility of BNP in HF management stems from its role in the body’s natural response to cardiac stress, promoting natriuresis (excretion of sodium in urine) and diuresis (increased urine production), which help to reduce blood volume and pressure, thus easing the strain on the heart. BNP levels increase proportionally to the severity of HF, making it a reliable biomarker for diagnosing and monitoring this condition. Additionally, BNP levels can provide prognostic information, as higher levels are associated with increased mortality and morbidity. The use of BNP testing in clinical practice aids in the early detection of HF, assessment of its severity, and ongoing evaluation of treatment efficacy [44,45,46,47].

#### 3.1.4. N-Terminal Pro-BNP (NT-proBNP)

NT-proBNP is a fragment of the prohormone BNP that is released into the blood alongside BNP in response to ventricular stretching and stress. Elevated NT-proBNP levels are indicative of HF and provide valuable information for diagnosis, risk stratification, and monitoring treatment efficacy. NT-proBNP is a reliable biomarker for assessing the severity of HF, as its levels correlate with the degree of ventricular dysfunction. One of the advantages of NT-proBNP over BNP is that its levels are less influenced by body mass index (BMI), making it a preferred marker in obese patients where BNP levels might be lower than expected [46,47,48]. NT-proBNP testing aids in differentiating HF from other causes of dyspnea, helping clinicians make accurate diagnoses and appropriate treatment decisions. The stability and longer half-life of NT-proBNP in the bloodstream also contribute to its reliability as a diagnostic tool. Additionally, serial measurements of NT-proBNP can be used to monitor the progression of HF and the effectiveness of therapeutic interventions, allowing for timely adjustments in treatment plans. Elevated NT-proBNP levels are associated with worse clinical outcomes, including higher rates of hospitalization and mortality, underscoring its prognostic value. NT-proBNP is an essential biomarker offering diagnostic, prognostic, and monitoring benefits. Its reduced sensitivity to BMI variations and its stability in the bloodstream make it a superior choice for assessing and managing HF in a wide range of patients [46,47,48,49,50].

#### 3.1.5. Mid-Regional Pro-Atrial Natriuretic Peptide (MR-proANP)

**Figure 8:**
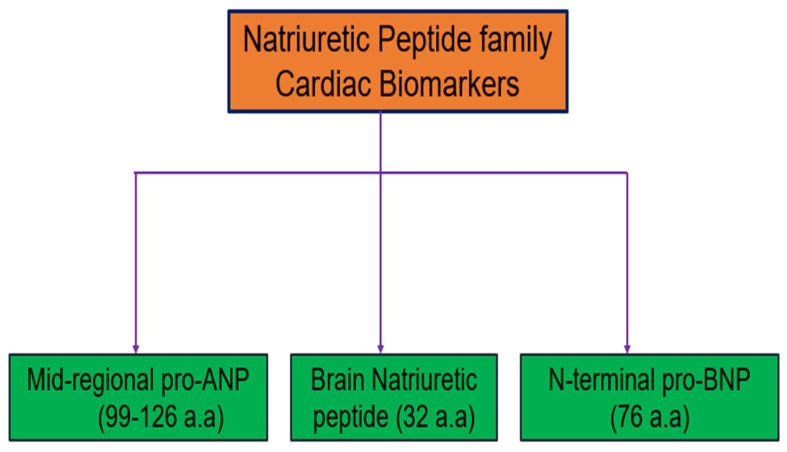
Natriuretic family cardiac biomarkers, which include MR-proANP, BNP, and NT pro-BNP. (Figure is created using app.biorender.com, accessed on 15 June 2024).

MR-proANP is a biomarker derived from the atrial natriuretic peptide (ANP) precursor, which is released from the atria of the heart in response to atrial stretching and volume overload. MR-proANP provides valuable diagnostic and prognostic information in CVDs, particularly HF. Elevated MR-proANP levels are indicative of increased cardiac wall stress and fluid overload, making it a useful marker for diagnosing acute and chronic HF (CHF). MR-proANP is beneficial because it is more stable than ANP in the bloodstream, allowing for more accurate and reliable measurements. This stability is crucial for clinical applications, ensuring consistent and reproducible test results [51,52]. The levels of MR-proANP correlate well with the severity of HF and are useful in risk stratification, helping to identify patients at higher risk of adverse outcomes. Additionally, MR-proANP can aid in the differentiation of HF from other causes of dyspnea, similar to BNP and NT-proBNP. Serial measurements of MR-proANP can be used to monitor disease progression and the effectiveness of treatment, guiding adjustments in therapeutic strategies. Elevated MR-proANP levels are associated with worse prognoses, including higher rates of hospitalization and mortality, emphasizing its role in the comprehensive management of HF patients. Thus, MR-proANP is a valuable cardiac biomarker for diagnosing and managing HF. Its stability and reliability make it an excellent tool for assessing cardiac stress and fluid overload, providing crucial information for diagnosis, risk stratification, and treatment monitoring [51,52,53]. Figure 8 represents different natriuretic family peptides, including BNP, NT-proBNP, and MR-proANP.

#### 3.1.6. Mid-Regional Pro-Adrenomedullin (MR-proADM)

MR-proADM is a stable fragment of the peptide hormone adrenomedullin, which is produced in various tissues, including the heart, lungs, and blood vessels. MR-proADM serves as a biomarker for assessing cardiovascular health and other conditions characterized by vascular dysfunction and inflammation. The hormone adrenomedullin has vasodilatory, anti-inflammatory, and natriuretic properties, playing a role in regulating blood pressure, fluid balance, and vascular tone. MR-proADM levels rise in response to increased cardiovascular stress, inflammation, and endothelial dysfunction. This makes it a useful marker for diagnosing and prognosticating HF, sepsis, and other conditions associated with systemic inflammation and vascular stress [53,54,55]. In HF, elevated MR-proADM levels are indicative of poor prognosis, correlating with higher risks of hospitalization and mortality. The measurement of MR-proADM can provide valuable information beyond traditional biomarkers, like BNP and NT-proBNP, especially in assessing the severity of illness and the risk of adverse outcomes. MR-proADM is particularly valuable in critical care settings, where it helps in the early identification of patients at risk of complications, such as sepsis or acute HF. It can also aid in monitoring the effectiveness of therapeutic interventions and guiding treatment adjustments, thus playing a crucial role in the comprehensive management of patients with acute and chronic conditions. MR-proADM is a significant biomarker that provides insights into cardiovascular health, vascular dysfunction, and systemic inflammation. Its stable nature and broad clinical applicability make it an important tool in diagnosing and managing HF, sepsis, and other conditions characterized by elevated cardiovascular and inflammatory stress [53,54,55,56,57].

#### 3.1.7. C-Reactive Protein (CRP)

CRP is a protein produced by the liver in response to inflammation in the body. Elevated levels of CRP in the blood are a general marker of inflammation and can indicate a higher risk of cardiovascular events [58]. High-sensitivity CRP (hs-CRP) testing is a more precise measurement that detects lower levels of CRP, allowing for the assessment of cardiovascular risk, particularly in individuals with intermediate risk. Systemic inflammation plays a significant role in the development and progression of atherosclerosis, the buildup of plaques in the arterial walls, which can lead to heart attacks and strokes. Elevated hs-CRP levels suggest the presence of such inflammation and can predict future cardiovascular events, even when cholesterol levels are normal [59]. Research has shown that individuals with higher hs-CRP levels are at an increased risk of heart disease, independent of other traditional risk factors. hs-CRP testing is especially useful in conjunction with other risk assessments, such as cholesterol levels and blood pressure measurements, to provide a more comprehensive evaluation of an individual’s cardiovascular risk. For example, individuals with intermediate risk for heart disease, as determined by traditional risk factors, can be further stratified based on their hs-CRP levels. Those with elevated hs-CRP may benefit from more aggressive preventive measures, such as lifestyle changes and medication. In addition to its role in assessing cardiovascular risk, hs-CRP can also be used to monitor the effectiveness of treatments aimed at reducing inflammation and cardiovascular risk. For instance, statins, which are commonly prescribed to lower cholesterol, also have anti-inflammatory effects and can reduce hs-CRP levels, providing a dual benefit in cardiovascular risk reduction [58,59,60].

#### 3.1.8. Heart-Type Fatty Acid-Binding Protein (H-FABP)

H-FABP is a small protein released into the bloodstream during myocardial injury, such as a heart attack. It rises rapidly, typically within 1–3 h after the onset of symptoms, making it valuable for early diagnosis of AMI. H-FABP levels peak around 6–8 h and return to baseline within 24 h, offering a timely window for detecting early cardiac events and ruling out acute episodes. Although H-FABP is less specific than cardiac troponins, as it can also be elevated in skeletal muscle injury and renal impairment, it serves as a useful complementary biomarker. Its rapid kinetics make H-FABP an effective tool in the early phase of AMI diagnosis, especially when combined with other markers, like troponins, for a more comprehensive assessment of myocardial injury [61,62]. Due to its high sensitivity for early detection, H-FABP can identify cardiac events earlier than troponins, making it particularly useful in the critical early hours following symptom onset. H-FABP is beneficial not only in emergency settings for early AMI detection but also in primary care and outpatient settings for ruling out cardiac events in patients with chest pain. Elevated H-FABP levels have been associated with worse outcomes in patients with acute coronary syndrome (ACS) and HF, adding prognostic value to its diagnostic capabilities. Beyond acute events, H-FABP may also provide insights into chronic cardiac conditions, including HF, where persistent elevations can indicate ongoing myocardial stress or injury [61,62,63].

#### 3.1.9. Myeloperoxidase (MPO)

MPO is an enzyme predominantly found in neutrophils and monocytes, playing a critical role in the body’s immune response by generating reactive oxygen species [64]. As a cardiac biomarker, MPO is significant due to its association with inflammation and oxidative stress, both of which are pivotal in the development and progression of cardiovascular diseases. Elevated MPO levels are indicative of increased risk for ACS and adverse CVD events. MPO contributes to the formation and destabilization of atherosclerotic plaques by oxidizing lipoproteins and promoting endothelial dysfunction, leading to plaque vulnerability and rupture. Additionally, MPO levels can aid in risk stratification and provide prognostic information for patients with chest pain, complementing other cardiac biomarkers, like troponins. Its utility extends to monitoring the effectiveness of therapeutic interventions aimed at reducing inflammation and oxidative stress, thereby enhancing the management of cardiovascular conditions [65,66,67]. However, it is important to note that the measurement of MPO is not routinely performed in clinical practice. This is due to various factors, including the availability of the assay, cost considerations, and the lack of standardized guidelines for its use in routine diagnostics. Additionally, the need for specialized equipment and the complexity of interpreting MPO levels in conjunction with other inflammatory markers contribute to its limited use. As such, while MPO holds significant potential as a biomarker, its use is primarily limited to research settings or specialized clinical cases. When measured, MPO is typically assessed in situations where there is a specific interest in understanding the role of inflammation and oxidative stress in cardiovascular pathology, often in the context of clinical trials or specialized cardiovascular research.

#### 3.1.10. Myoglobin

Myoglobin is an oxygen-binding protein found in heart and skeletal muscle tissues and is released into the bloodstream following muscle injury. As a cardiac biomarker, myoglobin is important due to its rapid release into the bloodstream within 1–3 h after the onset of myocardial infarction (MI), peaking around 6–9 h. This early rise makes myoglobin valuable for the early diagnosis of MI, enabling prompt medical intervention. However, myoglobin lacks specificity for cardiac muscle damage, as it can also be elevated in conditions involving skeletal muscle injury, such as trauma, strenuous exercise, or muscle diseases [68,69]. Its rapid clearance from the bloodstream, typically within 24 h, limits its utility for late diagnosis of MI. Despite these limitations, myoglobin is often used in conjunction with more specific biomarkers, like cardiac troponins, to enhance diagnostic accuracy. Its primary role lies in its ability to provide early indications of myocardial injury, which is crucial for the timely management of ACS [68,69,70].

#### 3.1.11. Ischemia-Modified Albumin (IMA)

IMA is a biomarker that signals myocardial ischemia, providing crucial information before myocardial necrosis occurs. Elevated IMA levels indicate the presence of ischemic conditions, typically rising rapidly within 1–2 h following the onset of myocardial ischemia [71]. This makes IMA valuable for the early detection of ACS, offering a preliminary warning that can prompt further diagnostic testing and intervention [72]. Unlike cardiac troponins, which detect myocardial damage, IMA identifies ischemic changes before significant tissue damage has occurred, allowing for timely intervention. However, IMA is less specific and can be influenced by conditions affecting the albumin molecule or other sources of ischemia. Despite this, its ability to provide early indications of ischemia is useful for triaging patients with chest pain and enhancing diagnostic accuracy when used alongside other cardiac biomarkers [43,71,72,73]. However, it is important to note that the measurement of IMA is not routinely performed in clinical practice. This is primarily due to the lack of widespread availability of the assay, variability in its diagnostic performance, and the influence of non-cardiac factors on its levels, which limits its routine application.

#### 3.1.12. Galectin-3

Galectin-3, a β-galactoside-binding lectin, is expressed by various immune cells, including mast cells, histiocytes, and macrophages. It plays a crucial role in diverse physiological functions. Galectin-3 is readily expressed on the cell surface and secreted by injured and inflammatory cells. Galectin-3 is a protein that plays a pivotal role in inflammation and fibrosis, with significant implications for HF pathology. Its elevated levels are not only associated with worse outcomes in HF but also serve as a critical biomarker for assessing disease severity and prognosis [74,75]. Galectin-3 aids in identifying patients at increased risk for disease progression and complications, thus guiding treatment strategies. Its involvement in myocardial fibrosis highlights its role in promoting adverse cardiac remodeling, which is central to chronic HF [76]. Beyond its prognostic value, galectin-3 levels can provide insight into the effectiveness of ongoing therapies and help tailor interventions based on individual patient responses. Additionally, galectin-3 may have utility in monitoring the impact of novel antifibrotic therapies, enhancing our ability to manage HF more effectively. Its integration with other biomarkers, such as BNP and NT-proBNP, offers a more nuanced understanding of cardiac health and disease dynamics [74,75,76,77].

#### 3.1.13. Copeptin

Copeptin, a peptide derived from the vasopressin precursor, is increasingly recognized as a valuable biomarker in the assessment of cardiovascular conditions and acute illnesses. It is released into the bloodstream during stress and acute events, reflecting neurohormonal activation and the body’s stress response. Elevated copeptin levels are closely associated with ACS, HF, and myocardial injury, providing crucial diagnostic and prognostic information [78]. Additionally, its role in acute ischemic stroke highlights its potential for broader applications in acute neurological conditions, further emphasizing its significance in risk stratification and management [79,80]. Notably, copeptin levels rise rapidly following myocardial injury, often preceding increases in traditional biomarkers, like troponins, which enhances its utility for early detection of myocardial infarction. Copeptin also offers advantages over other biomarkers due to its minimal influence on renal function and comorbidities, making it a more reliable marker in diverse patient populations. 

#### 3.1.14. Fibroblast Growth Factor 23 (FGF-23)

FGF-23 acts as a phosphaturic hormone, a circulating endocrine regulator of mineral metabolism, and a significant cardiac biomarker, and it is particularly noted for its role in chronic kidney disease (CKD) and HF. As a hormone involved in regulating phosphate and vitamin D metabolism, FGF-23 levels rise markedly in CKD patients. Elevated FGF-23 is closely associated with increased cardiovascular risk, predicting adverse outcomes such as left ventricular hypertrophy, HF, and vascular calcification [81]. Its prognostic value in HF patients is notable, with higher levels correlating with increased rates of hospitalization and mortality. FGF-23 also enhances risk stratification when used alongside established biomarkers, like BNP and NT-proBNP, offering a more nuanced understanding of a patient’s cardiovascular status. Moreover, elevated FGF-23 levels can indicate the progression of CKD and its cardiovascular complications, making it a crucial marker for disease severity and progression. Given its significant role in cardiovascular pathology, FGF-23 is being explored as a potential therapeutic target to mitigate cardiovascular risk in CKD patients [82,83].

#### 3.1.15. Growth Differentiation Factor-15 (GDF-15)

GDF-15 is a stress-responsive cytokine and systemic biomarker that reflects cellular aging and systemic inflammation. It has emerged as a significant indicator of CVD, with elevated levels linked to an increased risk of HF, myocardial infarction, and overall cardiovascular risk. In patients with coronary artery disease, atrial fibrillation, and HF, GDF-15 may provide additional prognostic information, offering valuable insights into patient outcomes. High levels of GDF-15 in HF patients, in particular, correlate with worse outcomes, making it an essential tool for risk stratification and guiding management decisions [84,85]. Its ability to reflect underlying pathophysiological processes such as inflammation, oxidative stress, and myocardial injury enhances its prognostic value. GDF-15 can be used in conjunction with other biomarkers, like BNP and NT-proBNP, to offer a comprehensive assessment of cardiac health. Its role in predicting long-term outcomes and monitoring disease progression and therapeutic response underscores its importance in personalized cardiovascular care, ultimately contributing to improved patient outcomes [84,85,86].

#### 3.1.16. Soluble ST2 (sST2)

ST2 (Suppression of Tumorigenicity 2) is a member of the IL-1 receptor family and exists in two forms: a transmembrane receptor (ST2L) and a soluble form (sST2), with the latter being particularly important as a cardiac biomarker. sST2 is a biomarker indicative of cardiac stress and remodeling and is particularly valuable in the management of HF. Elevated sST2 levels are strongly associated with worse outcomes in HF patients, making it essential for risk stratification and monitoring treatment efficacy. Unlike some biomarkers, sST2 provides dynamic information, reflecting real-time changes in disease status and response to therapy. When used alongside other biomarkers, like BNP and NT-proBNP, sST2 enhances the comprehensive assessment of cardiac health and understanding of disease progression [87,88].

#### 3.1.17. Endothelin-1 (ET-1)

Endothelin-1 (ET-1) is a potent vasoconstrictor peptide produced by endothelial cells, playing a crucial role in regulating vascular tone and blood pressure [89]. Elevated ET-1 levels are associated with hypertension, HF, and atherosclerosis, contributing to endothelial dysfunction and vascular remodeling. In hypertension, ET-1 increases peripheral resistance and blood pressure, while in HF, it promotes myocardial hypertrophy, fibrosis, and adverse cardiac remodeling. ET-1 also exacerbates atherosclerosis by reducing nitric oxide availability and promoting inflammation, influencing plaque stability and increasing the risk of cardiovascular events. ET-1 receptor antagonists are used in treating pulmonary arterial hypertension and have the potential to manage systemic hypertension and HF [90]. Measuring ET-1 levels aids in risk stratification and monitoring treatment response, enhancing personalized treatment approaches and optimizing cardiovascular outcomes. However, it is important to note that the measurement of ET-1 is not routinely performed in clinical practice. This is due to the complexity of the assay, the influence of various factors on ET-1 levels, and the limited availability of standardized tests, which restricts its use to research settings or specific clinical scenarios. ET-1 measurement is typically conducted in the context of specialized cardiovascular research or in clinical trials assessing the efficacy of ET-1 receptor antagonists. Additionally, it may be measured in patients with pulmonary arterial hypertension or other conditions where ET-1 plays a significant pathophysiological role.

#### 3.1.18. Cardiac Myosin-Binding Protein-C (cMyBP-C)

cMyBP-C plays a critical role in the regulation of sarcomere function within cardiomyocytes, contributing to the structural integrity and contractile performance of the heart muscle. As an emerging biomarker, cMyBP-C is gaining attention for its potential in the early diagnosis of myocardial infarction (AMI), where elevated levels are indicative of cardiac injury. This biomarker’s release into the bloodstream during acute myocardial injury offers a valuable window for early intervention. Furthermore, cMyBP-C levels may provide insights into the severity of myocardial damage, aiding in the risk stratification of patients and potentially guiding therapeutic decisions [91].

#### 3.1.19. Trimethylamine-N-Oxide (TMAO)

TMAO is a metabolite produced by gut microbiota through the metabolism of dietary choline and carnitine, commonly found in foods like red meat, eggs, and dairy products. Elevated levels of TMAO have been linked to an increased risk of cardiovascular events, including the development and progression of atherosclerosis and heart failure. The presence of high TMAO concentrations is thought to contribute to the promotion of vascular inflammation and the formation of arterial plaques, thereby exacerbating the risk of adverse cardiovascular outcomes [92].

#### 3.1.20. Adiponectin

Adiponectin is an anti-inflammatory adipokine primarily secreted by adipose tissue, playing a crucial role in regulating glucose levels and fatty acid breakdown. This hormone enhances insulin sensitivity and exerts protective effects on the cardiovascular system. Low levels of adiponectin are commonly associated with conditions such as obesity, insulin resistance, and an increased risk of cardiovascular diseases. Conversely, higher levels of adiponectin are considered cardioprotective, as they help to maintain vascular health by reducing inflammation and preventing the progression of atherosclerosis. Adiponectin’s role in metabolic syndrome and overall vascular health highlights its potential as a therapeutic target for preventing and managing cardiovascular and metabolic disorders [93,94].

#### 3.1.21. Interleukin-6 (IL-6)

IL-6 is a pro-inflammatory cytokine that plays a crucial role in the inflammatory response and immune system regulation. Elevated IL-6 levels are strongly associated with increased cardiovascular risk and adverse outcomes in HF patients. As a marker of systemic inflammation, IL-6 can help assess the degree of inflammation and cardiovascular risk in individuals with heart disease. Chronic elevation of IL-6 is linked to the progression of atherosclerosis and can contribute to the development and worsening of cardiovascular diseases. By providing insights into ongoing inflammatory processes, IL-6 testing aids in the risk assessment and management of HF and other cardiovascular conditions. Additionally, due to its role in inflammation, IL-6 is being explored as a potential target for novel anti-inflammatory therapies aimed at improving cardiovascular outcomes [95,96].

#### 3.1.22. Tumor Necrosis Factor-Alpha (TNF-α)

TNF-α is a key cytokine involved in systemic inflammation and the immune response. Elevated TNF-α levels are associated with HF and atherosclerosis, reflecting the underlying inflammatory processes that contribute to cardiovascular disease [97]. TNF-α plays a significant role in the pathogenesis of HF by promoting myocardial remodeling and dysfunction, which exacerbate cardiac impairment. Its levels correlate with disease severity and can provide valuable insights into the inflammatory state of patients with cardiovascular conditions. Given its central role in inflammation and myocardial injury, TNF-α is a potential target for anti-inflammatory therapies designed to mitigate cardiovascular damage and improve clinical outcomes [97,98].

#### 3.1.23. MicroRNAs (miRNAs) as Cardiac Biomarkers

**Figure 9:**
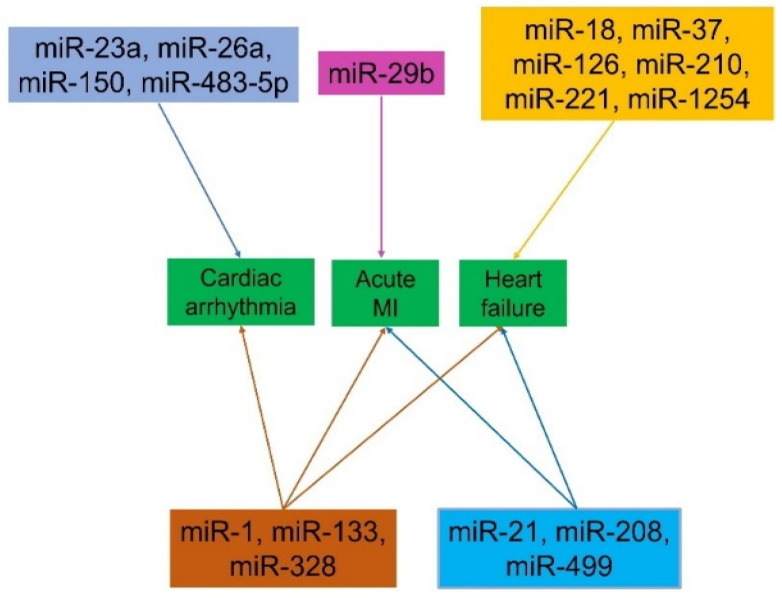
Specific and common miRNA biomarkers for various CVD conditions, including arrhythmia, heart failure, and acute myocardial infarction (MI). (Figure is created using app.biorender.com, accessed on 15 July 2024).

miRNAs are small, non-coding RNA molecules that play a critical role in gene regulation. Their unique expression profiles and release patterns during various stages of cardiovascular diseases (CVDs) make them valuable as novel biomarkers for diagnosis and prognosis. During myocardial injury and HF, specific miRNAs are released into the bloodstream, providing insight into the underlying molecular mechanisms and disease progression [99,100]. For AMI, miR-29b has been identified as a significant marker, while miR-1, miR-133, and miR-328 are relevant across AMI, arrhythmia, and HF. In cases of arrhythmia, miRNAs such as miR-23a, miR-26a, miR-150, and miR-483-5p are particularly indicative. For HF, miRNAs like miR-18, miR-37, miR-126, miR-210, miR-221, and miR-1254 offer diagnostic and prognostic value. Furthermore, miR-21, miR-208, and miR-499 are markers for both AMI and HF, enhancing the understanding of these conditions (Figure 9). The profiling of miRNAs provides detailed insights into the molecular mechanisms driving CVDs, revealing potential therapeutic targets and diagnostic tools. The use of miRNAs as biomarkers holds significant promise in advancing personalized medicine for cardiovascular health [101].

#### 3.1.24. Homocysteine 

**Figure 10:**
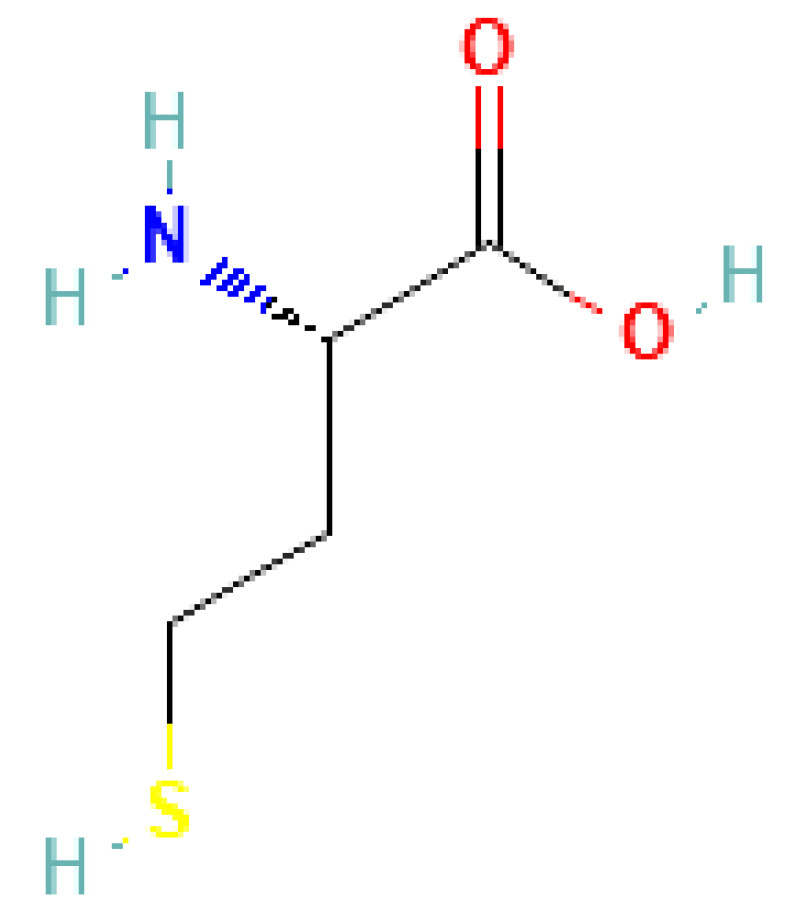
The structure of homocysteine (figure is retrieved from PubChem.ncbi.nlm.nih.gov, accessed on 12 June 2024).

Homocysteine is an amino acid (Figure 10) in the blood that, when elevated, is associated with endothelial dysfunction and an increased risk of cardiovascular disease. High levels of homocysteine can lead to atherosclerosis by damaging the inner lining of arteries and promoting blood clots. It serves as a marker for cardiovascular risk assessment, as elevated homocysteine levels are linked to conditions such as coronary artery disease, stroke, and peripheral arterial disease. Monitoring homocysteine levels can aid in identifying individuals at higher risk of cardiovascular events, allowing for early intervention and management strategies to mitigate these risks [102].

#### 3.1.25. Phospholipase A2 (PLA2)

PLA2 is an enzyme that hydrolyses phospholipids, releasing free fatty acids and lysophospholipids. Primarily secreted by immune cells, such as macrophages and neutrophils, PLA2 plays a crucial role in inflammation and the development of atherosclerosis. Elevated levels of PLA2 are associated with increased cardiovascular risk, as the enzyme contributes significantly to the formation and progression of atherosclerotic plaques. PLA2 promotes plaque formation by facilitating the accumulation of oxidized low-density lipoprotein (oxLDL) within arterial walls. It also contributes to plaque instability by weakening the fibrous cap that covers the plaque, making it prone to rupture. Additionally, PLA2-generated lysophospholipids can trigger platelet activation and thrombus formation, further increasing cardiovascular risk [103].

#### 3.1.26. Oxidized Low-Density Lipoprotein (oxLDL)

oxLDL indicates the oxidative modification of LDL particles, which play a crucial role in the initiation and progression of atherosclerosis. The presence of oxLDL triggers inflammatory responses and promotes the formation of foam cells, contributing to plaque buildup in the arteries. Macrophages take up OxLDL via scavenger receptors, transforming into foam cells within atherosclerotic plaques. OxLDL impairs endothelial function, leading to inflammation and the expression of adhesion molecules. Monitoring oxLDL levels helps in assessing oxidative stress and atherosclerotic burden, providing valuable insights into CVD risk and the effectiveness of antioxidant and lipid-lowering therapies [104]. 

#### 3.1.27. Lipoprotein (a) [Lp(a)]

Lipoprotein (a) [Lp(a)] is a variant of low-density lipoprotein (LDL) cholesterol that is particularly atherogenic due to its structural similarity to plasminogen, which can interfere with fibrinolysis and promote plaque formation. Elevated levels of Lp(a) are recognized as an independent risk factor for cardiovascular diseases, including atherosclerosis, myocardial infarction (MI), and stroke. Unlike traditional LDL cholesterol, Lp(a) levels are largely genetically determined and remain relatively constant throughout life, making them a crucial consideration in the assessment of cardiovascular risk [105].

#### 3.1.28. Apolipoproteins (ApoA1:ApoB)

Apolipoproteins, specifically ApoA1 and ApoB, are key components of lipoprotein particles that play significant roles in lipid metabolism and cardiovascular health. ApoA1 is the main protein component of high-density lipoprotein (HDL), which is involved in reverse cholesterol transport and is generally considered protective against cardiovascular disease. In contrast, ApoB is the primary protein of LDL and other atherogenic lipoproteins, contributing to the formation of arterial plaques. The ratio of ApoB to ApoA1 provides a more precise assessment of cardiovascular risk than traditional lipid measurements, as it directly reflects the balance between atherogenic and anti-atherogenic lipoproteins. A higher ApoB/ApoA1 ratio is associated with an increased risk of atherosclerosis and myocardial infarction, making it a valuable marker in the prediction and management of cardiovascular events [106].

#### 3.1.29. Oxylipins

Oxylipins are bioactive lipid mediators derived from the oxidation of polyunsaturated fatty acids, such as arachidonic acid, linoleic acid, and eicosapentaenoic acid. These compounds play a crucial role in modulating inflammation and vascular function, influencing processes like vasodilation, platelet aggregation, and leukocyte adhesion. Due to their involvement in these key physiological pathways, oxylipins have emerged as valuable biomarkers for detecting and monitoring cardiovascular diseases, including atherosclerosis and hypertension. Additionally, oxylipins are being studied for their potential to provide insights into the efficacy of therapeutic interventions aimed at reducing cardiovascular risk, offering a promising avenue for personalized medicine. Understanding the specific profiles of oxylipins in various cardiovascular conditions could lead to more targeted and effective treatment strategies [107,108].

**Figure 11:**
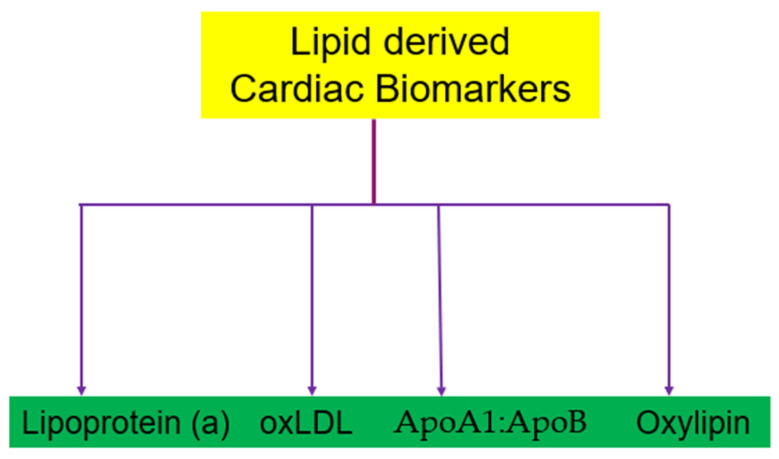
Lipid-derived cardiac biomarkers, such as lipoprotein (a), oxLDL, ApoA1:ApoB, and oxylipins.

Figure 11 represents lipid-derived cardiac biomarkers such as lipoprotein (a), oxLDL, apolipoproteins (ApoA1:ApoB), and oxylipins.

Cardiac biomarkers are essential for diagnosing and managing cardiovascular diseases, providing insights into disease mechanisms and progression. They help in risk assessment, early detection, and monitoring of treatment efficacy. Advanced research focuses on novel biomarkers, like microRNAs and inflammatory cytokines, offering the potential for more precise and personalized therapies. Table 2 provides a comprehensive overview of various biomarkers, highlighting their types, primary roles, diagnostic and prognostic utilities, and associated medical conditions. Future perspectives include integrating multi-biomarker approaches with advanced imaging and genomic data to enhance predictive accuracy and therapeutic outcomes in cardiovascular care.

### 3.2. Cardiac Imaging Modalities

#### 3.2.1. Chest X-ray

A chest X-ray is a fundamental imaging technique employed to visualize the heart, lungs, and chest wall. It is particularly useful for detecting heart enlargement, fluid accumulation in the lungs, and other chest abnormalities. Despite its ability to provide a broad overview of the chest, chest X-rays are limited in their capacity to deliver detailed information about the heart’s structures and function. This imaging modality is quick, widely accessible, and cost effective, making it a common initial diagnostic tool. However, its sensitivity is limited, particularly for identifying early or subtle cardiac changes, necessitating more advanced imaging techniques for comprehensive cardiac assessment [109,110].

#### 3.2.2. Electrocardiogram (ECG)

An ECG is a crucial diagnostic tool that records the electrical activity of the heart through electrodes placed on the skin. For the evaluation of the heart rate, ECGs measure heart rate accurately, whether it is normal, tachycardic, or bradycardic, aiding in the diagnosis of underlying causes of abnormal heart rates. To monitor cardiac conditions, regular ECGs are used to monitor patients with known cardiac conditions, such as HF or valvular heart disease, and assess disease progression and treatment efficacy. To assess heart size and function, ECGs can provide indirect information about heart chamber enlargement or hypertrophy, helping evaluate the impact of various conditions on cardiac structure [111,112]. To diagnose arrhythmias, ECGs are essential for detecting various arrhythmias, including atrial fibrillation, ventricular tachycardia, and bradycardias, by capturing deviations in heart rhythm and electrical conduction [111,112,113]. For the detection of myocardial infarction, ECGs play a vital role in diagnosing myocardial infarction (MI) by identifying ST segment elevation or depression, T-wave inversion, and abnormal Q waves, which indicate ischemic changes [114]. For the detection of electrolyte imbalances, ECGs can reveal abnormalities related to electrolyte imbalances, such as hyperkalemia or hypocalcemia, which affect cardiac electrical activity [115]. For the identification of conduction abnormalities, ECGs can identify various conduction disturbances, including bundle branch blocks and atrioventricular (AV) blocks, which can influence treatment decisions [116]. For the evaluation of pacemaker function, for patients with implanted pacemakers, ECGs are used to assess the proper functioning and pacing of the device, ensuring it meets the therapeutic goals [117]. For the assessment of myocarditis and pericarditis, ECGs can help diagnose inflammation of the heart muscle (myocarditis) or the lining around the heart (pericarditis) by revealing specific patterns of ST segment elevation or depression [118]. ECGs are relatively inexpensive compared to other imaging modalities, making them an accessible option for routine cardiac evaluations and emergency situations. While ECGs are indispensable for initial cardiac assessment and monitoring, they are sometimes complemented by other diagnostic tools for a complete evaluation of cardiac health.

#### 3.2.3. Echocardiography/Ultrasound

Echocardiography uses ultrasound waves to create detailed images of the heart’s structure and function. This non-invasive technique provides real-time images, allowing for dynamic assessment of heart size, shape, and pumping capacity [119]. Echocardiography is essential in diagnosing heart valve diseases, such as aortic stenosis, mitral regurgitation, and other valvular abnormalities. By assessing valve function and the severity of these conditions, echocardiography helps determine the appropriate timing for surgical or medical interventions [120]. Echocardiography is invaluable in diagnosing and monitoring cardiomyopathies, including hypertrophic, dilated, and restrictive types. It provides detailed information on ventricular wall thickness, chamber size, and systolic and diastolic function, enabling tailored treatment strategies [121,122]. For congenital heart defects, echocardiography offers precise anatomical details that are crucial for diagnosis and surgical planning. It is particularly useful in pediatric cardiology, providing a non-invasive means to monitor heart development and detect abnormalities early in life [123,124]. In ischemic heart disease, echocardiography can detect regional wall motion abnormalities that are indicative of myocardial infarction or ischemia [125]. Stress echocardiography evaluates how the heart responds to stress, offering insights into coronary artery disease and guiding revascularization decisions [126]. Advancements in echocardiographic technology, such as three-dimensional echocardiography, enhance diagnostic accuracy by providing more detailed and comprehensive views of cardiac structures [127]. Contrast-enhanced echocardiography improves the visualization of cardiac structures and functions, particularly in patients where traditional imaging is limited by body habitus or lung interference [128]. Despite its many advantages, the quality of echocardiographic images can be affected by factors, such as the patient’s body habitus and lung interference, which may obscure or distort the ultrasound waves. Echocardiography remains a cornerstone of cardiac diagnostics, offering comprehensive, real-time insights into cardiac health. Its applications in diagnosing and monitoring various cardiac conditions make it an indispensable tool in the management of cardiovascular diseases, enhancing patient care and outcomes.

#### 3.2.4. Computed Tomography (CT) Scan

A Computed Tomography (CT) scan uses X-rays to create detailed cross-sectional images of the heart and blood vessels. This imaging technique is invaluable in diagnosing and assessing various cardiovascular conditions, providing high-resolution images that enable accurate evaluation of heart structures and blood flow [129]. CT scans can help in the detailed assessment of congenital heart defects, providing precise anatomical information that is essential for surgical planning [130]. CT imaging can identify and characterize cardiac tumors, aiding in the diagnosis and management of these rare conditions [131]. CT scans are highly effective in detecting aortic aneurysms and monitoring their size over time [132]. CT angiography (CTA) is particularly useful for detecting coronary artery disease (CAD). It allows for the visualization of coronary arteries to identify blockages or narrowing. Additionally, CTA is highly sensitive for excluding coronary artery disease, making it a valuable diagnostic tool for patients with low to intermediate risk of CAD. By providing detailed images of the coronary vessels, CTA helps in the non-invasive assessment of coronary artery stenosis and guides further treatment decisions [133]. A specific type of CT scan, called a coronary calcium scan, measures the amount of calcium in the coronary arteries. The presence of calcium deposits is a marker of atherosclerosis and helps in risk stratification for future cardiac events [134]. CT pulmonary angiography (CTPA) is the gold standard for diagnosing pulmonary embolisms, providing detailed images of the pulmonary arteries to detect blood clots [135]. The CT Fractional Flow Reserve (FFR-CT) technique uses CT images to non-invasively measure the blood flow and pressure in the coronary arteries, helping to determine the physiological impact of coronary stenoses [136]. Dual-energy CT can differentiate between different tissue types, improving the visualization of plaque composition and enhancing the assessment of myocardial perfusion [137]. An advantage of CT scans is that they provide high-resolution images, allowing for precise anatomical evaluation. CT scans are relatively quick, making them suitable for emergency situations where rapid diagnosis is essential. A Disadvantage of CT scans is that involve exposure to ionizing radiation, which carries a risk of cancer, particularly with repeated imaging. The use of contrast agents can be problematic for patients with kidney issues or allergies, as these agents can cause nephropathy or allergic reactions. While CT provides excellent anatomical detail, it offers limited functional information compared to other imaging modalities, like MRI or echocardiography [129].

#### 3.2.5. Positron Emission Tomography (PET) 

A PET scan uses radioactive tracers to assess the metabolic activity of heart tissues. This imaging modality is invaluable in evaluating myocardial perfusion, viability, and inflammation, providing detailed functional information that is crucial for diagnosing and managing various cardiac conditions. PET scans are highly effective in assessing myocardial perfusion, which is the blood flow to the heart muscle. This helps in identifying areas with reduced blood flow, indicative of coronary artery disease [138]. PET scans can distinguish between viable and non-viable heart tissue, particularly after a myocardial infarction. This information is critical in determining the potential for functional recovery of the heart muscle and guiding revascularization decisions [139]. PET imaging is useful in detecting inflammation and infection in conditions, like cardiac sarcoidosis and myocarditis, providing insights into disease activity and guiding treatment [140]. PET scans help in detecting coronary artery disease and assessing the severity and extent of ischemia. This is essential for risk stratification and treatment planning. PET scans are used to monitor the effects of treatments, such as revascularization procedures or medical therapies, by evaluating changes in myocardial perfusion and metabolism. Combining PET with CT or MRI (PET/CT and PET/MRI hybrid imaging modalities) enhances anatomical localization and provides comprehensive information on both structure and function. This hybrid imaging is particularly useful in complex cases requiring detailed assessment [141]. PET allows for the absolute quantification of myocardial blood flow, providing precise measurements that are important for accurate diagnosis and management [142]. Advantages include high sensitivity and specificity in detecting metabolic changes in heart tissues. PET provides detailed functional information, including blood flow, tissue viability, and metabolic activity. The use of radioactive tracers involves radiation exposure, which carries inherent risks. PET scanners are expensive and have limited availability. PET scans require specific preparation, including fasting and tracer administration, and they are time consuming.

#### 3.2.6. Single-Photon Emission Computed Tomography (SPECT) 

SPECT scan uses gamma rays to create three-dimensional images of the heart, offering crucial insights into myocardial blood flow and cardiac function. SPECT scans are often used in conjunction with stress tests to assess how well blood flows through the heart during physical exertion compared to rest. SPECT imaging helps in diagnosing CAD and assessing the extent and severity of ischemia, which is crucial for risk stratification and management [143,144]. SPECT can help determine which areas of the heart muscle are still viable and capable of recovery after a myocardial infarction. This is important for planning revascularization procedures [145]. SPECT scans provide valuable information on left ventricular function, including ejection fraction, which is important for evaluating HF and guiding therapy [146]. Gated SPECT combines traditional SPECT imaging with electrocardiographic gating to provide detailed information on both myocardial perfusion and ventricular function, including wall motion and thickening [147]. Combining SPECT with CT (SPECT/CT hybrid imaging) enhances anatomical localization and provides comprehensive information on both perfusion and structural abnormalities [148]. Compared to PET scans, SPECT provides lower spatial resolution, which can limit the detail and accuracy of the images. The procedure can be time consuming, requiring preparation, such as fasting and the administration of radioactive tracers.

#### 3.2.7. Magnetic Resonance Imaging (MRI)

MRI uses strong magnetic fields and radio waves to create detailed images of the heart and blood vessels. This non-invasive imaging technique is highly valuable in diagnosing and evaluating a wide range of cardiovascular conditions [149]. MRI is crucial for diagnosing various cardiomyopathies, such as hypertrophic and dilated types. It provides detailed images of heart muscle thickness, structure, and function [150]. MRI is used to diagnose and assess congenital heart defects, offering precise images that are essential for planning surgical or interventional procedures [151]. MRI helps in identifying areas of myocardial infarction, distinguishing between viable and non-viable tissue, and assessing the extent of scarring. This information is critical for determining treatment strategies [152]. MRI is effective in detecting inflammation of the heart muscle (myocarditis) and the lining around the heart (pericarditis), providing insights into disease activity and guiding therapy [153]. MRI provides detailed images of heart valves, helping in the assessment of valvular heart diseases, including stenosis and regurgitation [154]. MRI can measure blood flow through the heart and vessels, providing important data on cardiac output and shunt quantification in congenital heart disease. Stress cardiac MRI evaluates myocardial perfusion under stress conditions, similar to stress tests with SPECT or PET, aiding in the detection of ischemia and assessment of coronary artery disease [155]. T1 mapping is an advanced technique that assesses diffuse myocardial fibrosis, which is important for evaluating conditions, such as hypertensive heart disease and cardiomyopathies [156]. Late Gadolinium Enhancement (LGE) is a technique that allows for the detailed assessment of myocardial viability and scar tissue, which is important for evaluating the potential for recovery and guiding revascularization decisions [157]. MRI offers detailed functional and anatomical information, aiding in comprehensive cardiac evaluation. The advantages of MRI are that it provides high-resolution images with excellent soft tissue contrast, enabling detailed evaluation of cardiac structures. MRI does not involve ionizing radiation, making it safer for repeated use. Disadvantages include it being an expensive and lengthy procedure with limited availability. MRI may not be suitable for patients with certain implants, such as pacemakers or defibrillators, and those with severe claustrophobia. Despite its high cost, time-consuming nature, and contraindications for certain patients, MRI remains the best option and is a crucial modality for detailed cardiac evaluation, contributing significantly to improved diagnosis and patient care. 

Further, Table 3 provides the key aspects of various cardiac imaging modalities, including their principles, applications, advantages, and disadvantages. These advanced diagnostic tools, including cardiac biomarkers and imaging techniques, are essential for accurately diagnosing and managing cardiovascular diseases, ensuring timely and effective treatment for patients.

## 4. Essential Medications for Treating CVDs

Effective management of CVDs often requires a combination of lifestyle changes and pharmacotherapy. Medications play a crucial role in controlling symptoms, preventing disease progression, and reducing the risk of complications, such as heart attacks and strokes. This study presents some key medications used in the treatment of various CVDs.

### 4.1. Statins

**Figure 12:**
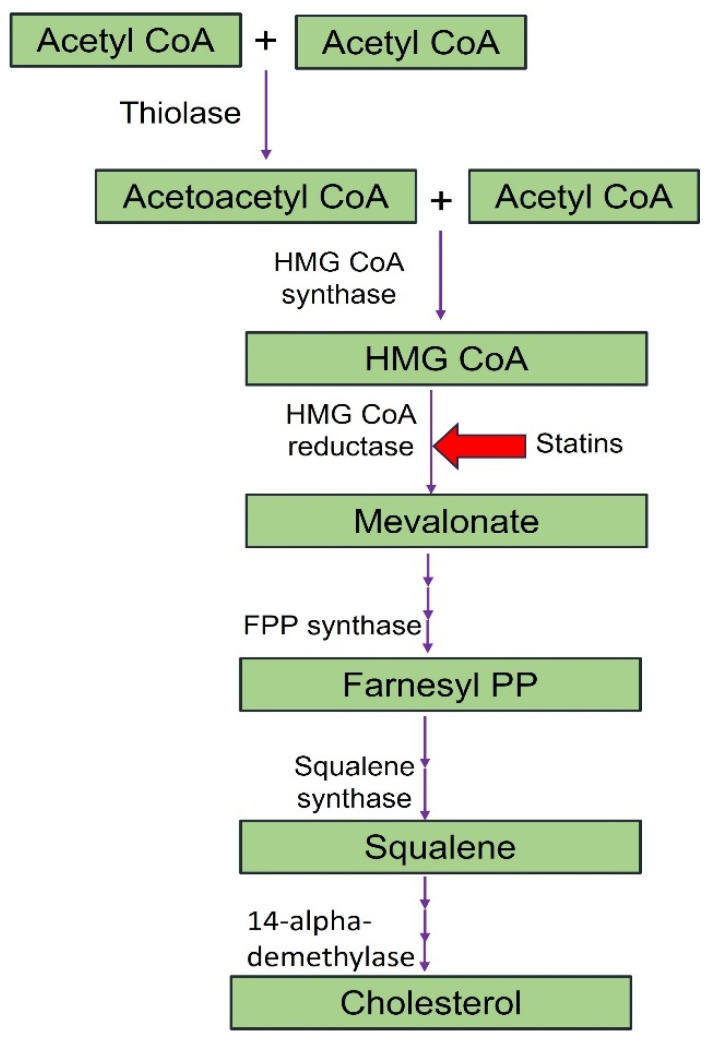
The inhibition of HMG CoA reductase by statins, which, in turn, leads to the inhibition of cholesterol synthesis. (Figure is created using app.biorender.com, accessed on 15 June 2024).

Statins (e.g., atorvastatin, fluvastatin) are used to lower cholesterol levels by inhibiting the enzyme HMG-CoA reductase (Figure 12), which plays a key role in cholesterol production in the liver. This reduction in cholesterol levels helps prevent the buildup of plaques in the arteries, reducing the risk of atherosclerosis. By lowering LDL cholesterol and total cholesterol, statins significantly decrease the risk of heart attacks, strokes, and peripheral artery disease [158]. Additionally, these medications help stabilize atherosclerotic plaques, decreasing the risk of plaque rupture and subsequent cardiovascular events. They also enhance endothelial function, which contributes to overall vascular health. Beyond their lipid-lowering capabilities, statins are believed to mitigate cardiovascular disease development through their antioxidant, anti-inflammatory, endothelial function-enhancing, plaque-stabilizing, and platelet aggregation-reducing properties [159,160,161]. 

### 4.2. Anticoagulants

Anticoagulants prevent blood clots by interfering with the coagulation cascade and are used for conditions, like atrial fibrillation and deep vein thrombosis. By thinning the blood, these medications reduce the risk of clot formation in the heart, veins, and arteries, thus preventing potentially life-threatening events, like pulmonary embolism (PE) and stroke [162,163]. Warfarin and newer agents, like rivaroxaban and apixaban, are common anticoagulants. Anticoagulants are essential in managing patients with mechanical heart valves, ensuring that clots do not form on the artificial valve surfaces. They also play a crucial role in the prevention and treatment of venous thromboembolism (VTE), which includes both deep vein thrombosis (DVT) and PE [164,165]. The newer direct oral anticoagulants (DOACs), like rivaroxaban and apixaban, offer the advantage of fewer dietary restrictions and do not require regular monitoring, like warfarin, making them more convenient for patients [166]. Anticoagulants are also used in the management of certain hypercoagulable states and for stroke prevention in patients with non-valvular atrial fibrillation [167]. 

### 4.3. Antiplatelet Agents

Antiplatelet agents are crucial in preventing platelets from clumping together to form blood clots, thus reducing the risk of heart attacks and strokes. They are essential in managing acute coronary syndrome (ACS) and in preventing recurrent cardiovascular disease (CVD) events in patients with a history of heart attack or stroke. Additionally, anti-platelet therapy is important in preventing clot formation in patients with stents or other cardiac interventions, ensuring the patency of these devices [168,169]. Aspirin and clopidogrel are widely used antiplatelet agents, often prescribed in combination for enhanced protective effects. Ticagrelor and prasugrel are newer antiplatelet agents that provide more potent platelet inhibition compared to clopidogrel. Ticagrelor offers reversible binding to the P2Y12 receptor, enhancing its efficacy in ACS management, while prasugrel provides a more consistent antiplatelet effect through irreversible binding, which is beneficial in reducing cardiovascular events during percutaneous coronary interventions. These agents also play a key role in secondary prevention for patients with atherosclerosis, significantly reducing morbidity and mortality. They are valuable in managing peripheral artery disease (PAD) by improving blood flow and reducing the risk of limb ischemia. For patients with atrial fibrillation at risk of stroke but unable to take anticoagulants, antiplatelet therapy may offer an alternative protective strategy [170,171,172].

### 4.4. ACE Inhibitors (Angiotensin-Converting Enzyme Inhibitors)

**Figure 13:**
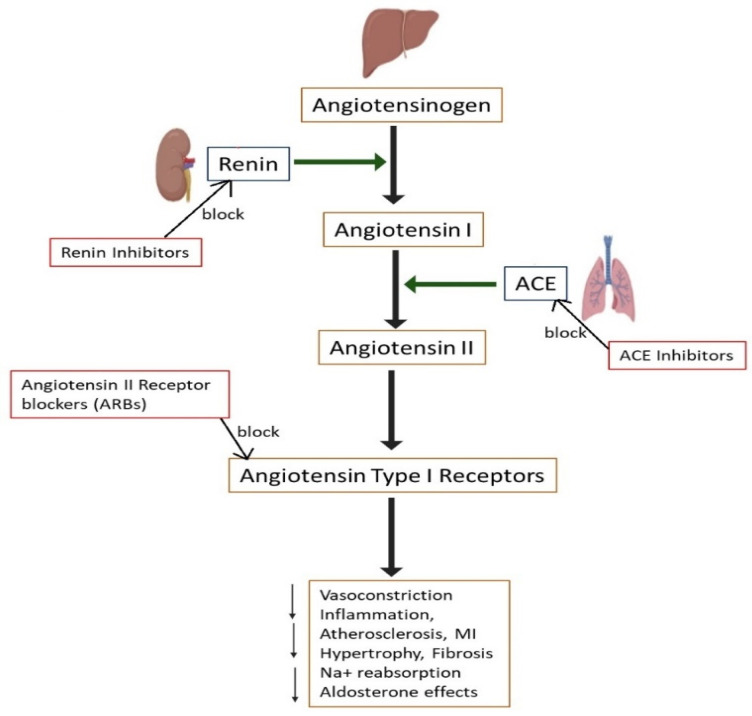
A schematic representation of mechanisms of both ACE inhibitors and ARBs. ACE inhibitors block the conversion of angiotensin I to angiotensin II, while ARBs directly block the action of angiotensin II by binding to its AT1 receptors. (Figure is created using app.biorender.com, accessed on 15 June 2024).

ACE inhibitors lower blood pressure by inhibiting the conversion of angiotensin I to angiotensin II, a potent vasoconstrictor. This results in vasodilation and reduced blood volume, lowering blood pressure and decreasing the workload on the heart. ACE inhibitors are also beneficial for patients with HF and chronic kidney disease due to their protective effects on the heart and kidneys. Examples include lisinopril and enalapril. Additionally, ACE inhibitors help prevent the remodeling of the heart and blood vessels, which can occur after a heart attack or in chronic hypertension, thereby improving long-term cardiovascular outcomes [173,174]. ACE inhibitors reduce systemic vascular resistance without raising heart rate and promote natriuresis. These drugs are highly effective in managing high blood pressure, lowering mortality rates in congestive HF and left ventricular dysfunction following a heart attack, and slowing the progression of diabetic kidney disease [175]. Furthermore, ACE inhibitors have been shown to improve endothelial function and reduce inflammation, contributing to overall cardiovascular health [176]. Figure 13 clearly illustrates the mechanism of ACE inhibitors. 

### 4.5. Angiotensin II Receptor Blockers (ARBs)

ARBs block the action of angiotensin II by binding to its AT1 (Angiotensin II Type 1) receptors on blood vessels, preventing vasoconstriction and lowering blood pressure. By relaxing blood vessels, ARBs improve blood flow and reduce the strain on the heart [177]. They are often used in patients who cannot tolerate ACE inhibitors and are effective in treating hypertension, heart failure (HF), and chronic kidney disease. Losartan and valsartan are common ARBs [178]. Additionally, ARBs decrease the overall vascular resistance and improve arterial compliance, which help reduce high blood pressure and thereby lower the risk of stroke and myocardial infarction. They also help to prevent the progression of left ventricular hypertrophy, a common consequence of chronic high blood pressure, thereby preserving cardiac function [179]. Furthermore, ARBs have a favorable side effect profile with a lower incidence of cough and angioedema compared to ACE inhibitors, making them a suitable alternative for many patients [180]. Figure 13 illustrates how ARBs block the action of angiotensin II by binding to its AT1 receptors. This leads to a reduction in vasoconstriction, inflammation, atherosclerosis, myocardial infarction, hypertrophy, and cardiac fibrosis.

### 4.6. Antiarrhythmic Agents

These are medications used to restore and maintain normal heart rhythm by modifying the electrical activity of the heart. 

#### 4.6.1. Class I Agents (Sodium Channel Blockers)

Class I antiarrhythmic agents work by blocking sodium channels in cardiac cells, which stabilizes the cardiac cell membrane and reduces the speed of electrical conduction. This action helps manage various types of arrhythmias by slowing down abnormal electrical signals and restoring normal rhythm [181,182]. Quinidine is often used in the treatment of atrial fibrillation and ventricular arrhythmias, and quinidine helps by decreasing the excitability of cardiac cells and prolonging the refractory period, thus reducing the frequency of arrhythmic events [183]. Procainamide is employed in the management of ventricular arrhythmias and atrial arrhythmias. It works by decreasing the rate of depolarization, which helps normalize the heart’s rhythm and prevent the reoccurrence of arrhythmias [184]. Flecainide, known for its potency, is used primarily for the management of atrial flutter and atrial fibrillation. It stabilizes the cardiac membrane by blocking sodium channels, which helps control rapid heart rates and prevent the propagation of abnormal impulses [185,186].

#### 4.6.2. Class II Agents (β-Blockers)

β-blockers reduce blood pressure and heart rate by blocking the effects of adrenaline on the heart and blood vessels, thereby managing hypertension and HF. They help decrease the heart’s demand for oxygen by slowing down the heartbeat and reducing its force of contraction. This makes β-blockers particularly useful for treating conditions, like angina, post-heart attack management, and arrhythmias, such as atrial fibrillation [187,188]. Common examples are metoprolol and propranolol. Additionally, β-blockers improve survival rates in patients who have experienced a myocardial infarction by reducing the risk of subsequent heart attacks. They also help to prevent sudden cardiac death by stabilizing the electrical activity of the heart and reducing the incidence of life-threatening arrhythmias. In patients with HF, β-blockers can improve symptoms and reduce hospitalizations by enhancing the heart’s efficiency and decreasing its workload. β-blockers are also beneficial in managing conditions associated with high sympathetic nervous system activity, such as anxiety and certain types of tremors [189,190].

#### 4.6.3. Class III Agents (Potassium Channel Blockers) 

Class III antiarrhythmic agents primarily work by blocking potassium channels, which prolongs the repolarization phase of the cardiac action potential. This prolongation helps stabilize the heart’s electrical activity and prevent the reoccurrence of arrhythmias [191]. Amiodarone, one of the most widely used antiarrhythmic drugs, is effective in treating a broad range of arrhythmias, including atrial fibrillation and ventricular tachycardia [192]. It prolongs the action potential duration and refractory period by inhibiting potassium channels, as well as affecting sodium, calcium, and beta-adrenergic receptors. Despite its efficacy, amiodarone can have significant side effects, including pulmonary toxicity and thyroid dysfunction [193]. Sotalol is used to manage both atrial and ventricular arrhythmias. It primarily works by blocking potassium channels, which prolongs the cardiac action potential and helps stabilize heart rhythm. Sotalol is also known for its beta-blocking properties, which contribute to its antiarrhythmic effects [194,195].

#### 4.6.4. Class IV Agents (Calcium Channel Blockers)

Calcium channel blockers (CCBs) relax and widen blood vessels by inhibiting calcium ions from entering cardiac and smooth muscle cells, thus reducing blood pressure. These medications also help in reducing the heart’s workload and are particularly effective in treating hypertension, angina, and certain arrhythmias [196]. They are divided into dihydropyridines (e.g., amlodipine and nifedipine), which primarily affect blood vessels, and non-dihydropyridines (e.g., diltiazem and verapamil), which also affect the heart rate. Additionally, CCBs improve myocardial oxygen delivery by dilating coronary arteries, which is particularly beneficial in treating chronic stable angina and vasospastic angina. In patients with atrial fibrillation, non-dihydropyridine CCBs, like verapamil and diltiazem, are effective in controlling ventricular rate. CCBs are often used in combination with other agents, such as ACE inhibitors or β-blockers, to achieve better blood pressure control [197,198,199,200,201].

These antiarrhythmic agents are critical in the management of arrhythmias, providing effective treatment options to restore and maintain normal heart rhythms. Their use is guided by the specific type of arrhythmia, patient characteristics, and potential side effects, with careful monitoring often required to ensure efficacy and safety.

### 4.7. Fibrates

Fibrates lower triglyceride levels and increase HDL cholesterol and are used to treat dyslipidemia. They work by activating peroxisome proliferator-activated receptors (PPARs), which regulate lipid metabolism, leading to decreased triglyceride production and increased clearance from the blood. Fenofibrate and gemfibrozil are commonly used fibrates, particularly in patients with high triglyceride levels and low HDL cholesterol. Additionally, fibrates can help reduce the risk of cardiovascular events by improving the overall lipid profile, particularly in individuals who have not achieved adequate lipid control with statins alone [202,203]. They may also have beneficial effects on lipoprotein composition and reduce the risk of pancreatitis in patients with severe hypertriglyceridemia. Fibrates are often used in combination with statins for enhanced lipid-lowering effects, though care must be taken to monitor for potential interactions and side effects, such as myopathy or liver enzyme elevations [204,205]. 

### 4.8. Neprilysin Inhibitors

Neprilysin inhibitors, when combined with angiotensin II receptor blockers (ARBs), offer a potent therapeutic approach for managing heart failure (HF). Neprilysin is an enzyme that breaks down natriuretic peptides, which are hormones that promote vasodilation, natriuresis (sodium excretion), and diuresis (fluid excretion). By inhibiting neprilysin, these medications enhance the effects of natriuretic peptides, thereby improving symptoms of HF and slowing disease progression [206,207]. The combination of neprilysin inhibition with ARB therapy provides a synergistic effect, as ARBs further reduce the effects of angiotensin II, another hormone that contributes to HF through vasoconstriction and fluid retention [208,209]. A notable example is the combination of sacubitril with valsartan, marketed as Entresto. Clinical trials have demonstrated that Entresto, which combines sacubitril with valsartan, significantly improves outcomes in patients with HF with reduced ejection fraction (HFrEF), including reductions in hospitalization rates and mortality [208,209,210]. Neprilysin inhibitors, when combined with ARB valsartan, offer a potent therapeutic approach for managing heart failure (HF). Sacubitril–valsartan is a cornerstone treatment for patients with HFrEF, not a second-line therapy, and it has been shown to significantly improve outcomes, including reductions in hospitalization rates and mortality.

### 4.9. Aldosterone Antagonists

Aldosterone antagonists block the effects of aldosterone, a hormone that causes salt and water retention, thus reducing blood pressure and alleviating symptoms of HF. By inhibiting aldosterone, these medications promote the excretion of sodium and water while retaining potassium, which is crucial for maintaining electrolyte balance and managing conditions like HF and resistant hypertension [211,212]. Aldosterone antagonists also help reduce myocardial fibrosis and ventricular remodeling, which can improve long-term cardiac function and outcomes. They are commonly used in combination with other medications, such as ACE inhibitors or β-blockers, to enhance therapeutic efficacy in managing CHF and post-myocardial infarction. Spironolactone and eplerenone are frequently prescribed examples, with eplerenone offering a more selective profile and potentially fewer side effects [212,213,214].

### 4.10. Diuretics

Diuretics help remove excess fluid from the body by increasing urine production, thereby reducing blood pressure and alleviating symptoms of HF. By decreasing the volume of fluid in the blood vessels, diuretics reduce the burden on the heart and lower the risk of fluid buildup in the lungs and other tissues. This fluid reduction helps manage symptoms like edema, pulmonary congestion, and ascites. There are different types of diuretics, each with specific indications and mechanisms of action. Loop diuretics, such as furosemide, are highly effective in rapidly removing fluid and are often used in acute settings or severe cases of HF. Thiazide diuretics, like hydrochlorothiazide, are generally used for long-term management of hypertension and mild fluid retention [215,216].

### 4.11. Nitrates

Nitrates dilate blood vessels, improving blood flow to the heart and relieving angina (chest pain). They work by releasing nitric oxide, which relaxes the smooth muscle in the vessel walls, allowing more blood and oxygen to reach the heart muscle. Nitrates are used both for immediate relief of angina symptoms and long-term management of chronic stable angina [217]. Examples include nitroglycerin and isosorbide dinitrate. Additionally, nitrates can reduce the heart’s workload by decreasing the volume of blood returning to the heart (preload), which alleviates the strain on the heart muscle. This preload reduction is particularly beneficial in managing conditions like HF [218].

### 4.12. Sodium–Glucose Cotransporter-2 Inhibitors (SGLT2 Inhibitors)

SGLT2 inhibitors, originally developed for the management of diabetes, have emerged as valuable treatments for HF and CVDs. These medications work by inhibiting SGLT2 in the kidneys, which reduces glucose reabsorption and increases glucose excretion in the urine, thereby lowering blood sugar levels [219]. Additionally, SGLT2 inhibitors promote diuresis, which helps reduce fluid overload and alleviate symptoms of HF. This diuretic effect leads to decreased blood volume and reduced cardiac stress, improving outcomes in HF patients. Drugs like empagliflozin and dapagliflozin have demonstrated significant cardiovascular benefits in clinical trials, including reductions in HF hospitalizations, cardiovascular death, and all-cause mortality [220,221].

### 4.13. Proprotein Convertase Subtilisin/Kexin Type 9 (PCSK9) Inhibitors

PCSK9 inhibitors lower LDL cholesterol levels by targeting and inhibiting the protein PCSK9, which plays a crucial role in regulating LDL cholesterol metabolism. PCSK9 binds to LDL receptors on liver cells, leading to their degradation and reducing the liver’s ability to clear LDL cholesterol from the blood. By inhibiting PCSK9, these medications increase the number of available LDL receptors, thereby enhancing the liver’s capacity to remove LDL cholesterol from the bloodstream. This results in significantly lower LDL cholesterol levels and provides an effective alternative for patients who do not achieve adequate cholesterol control with statins alone. PCSK9 inhibitors, such as alirocumab and evolocumab, have demonstrated substantial reductions in LDL cholesterol and have been shown to lower the risk of cardiovascular events, including heart attacks and strokes [222,223,224].

Thrombolytics, such as the tissue plasminogen activator (tPA), are crucial for the management of AMI and ischemic stroke. These medications work by dissolving blood clots that obstruct blood flow in coronary or cerebral arteries, thereby restoring circulation and minimizing tissue damage. Thrombolytic therapy is also recommended for managing VTE, including DVT and PE. The effectiveness of thrombolytics is highest when administered shortly after symptom onset, emphasizing the importance of rapid treatment to improve outcomes and reduce the risk of long-term complications [225,226].

### 4.14. Direct Renin Inhibitors

Direct renin inhibitors, like aliskiren, offer a novel approach to managing hypertension and HF by directly inhibiting the enzyme renin. This action prevents the formation of angiotensin I, which leads to reduced production of angiotensin II—a key player in the renin–angiotensin–aldosterone system (RAAS). By lowering blood pressure and alleviating stress on the heart, these inhibitors are beneficial for patients with resistant hypertension and can be used in conjunction with other antihypertensive therapies for enhanced efficacy [227,228].

### 4.15. Cholesterol Absorption Inhibitors

Ezetimibe lowers LDL cholesterol levels by blocking the absorption of cholesterol in the intestines. This mechanism helps to reduce overall cholesterol levels and is particularly effective when used in combination with statins. By decreasing intestinal cholesterol absorption, ezetimibe enhances lipid control, reducing the risk of cardiovascular events, such as heart attacks and strokes, especially in patients who do not achieve their cholesterol targets with statins alone [229,230]. 

### 4.16. Antisense Oligonucleotides

Inclisiran represents a newer class of medication that reduces PCSK9 levels through RNA-based mechanisms. This reduction enhances LDL cholesterol clearance from the blood, offering significant cardiovascular benefits. Inclisiran is a valuable addition to lipid-lowering therapies, especially for patients who need further LDL cholesterol reduction beyond what statins can achieve [231,232].

### 4.17. Ivabradine

Ivabradine is used to manage CHF by selectively lowering the heart rate without affecting blood pressure or myocardial contractility. It inhibits the If current in the sinoatrial node, which slows the heart rate and helps alleviate symptoms of HF. This selective action improves exercise tolerance and quality of life in patients, particularly those who remain symptomatic despite optimal beta-blocker therapy [233,234].

### 4.18. Ranolazine 

Ranolazine is effective in treating chronic angina by improving myocardial blood flow and reducing angina symptoms. It works by inhibiting the late sodium current in heart cells, which reduces intracellular calcium overload and enhances myocardial relaxation. Ranolazine can be used in conjunction with other anti-anginal medications to better control symptoms and reduce the frequency of angina attacks [235,236].

**Figure 14:**
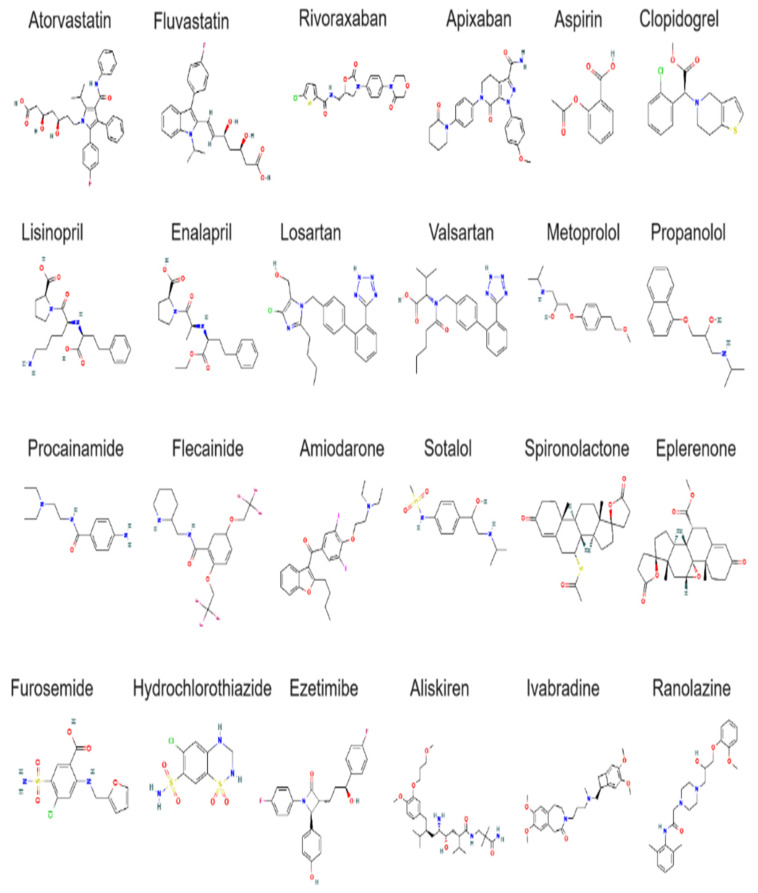
Different chemical inhibitors for effective management of CVDs. Structures were retrieved from PubChem.com, accessed on 15 June 2024.

Overall, effective management of CVDs often necessitates the use of a variety of medications. Statins lower cholesterol levels and stabilize atherosclerotic plaques, while anticoagulants and antiplatelet agents prevent blood clots, reducing the risk of heart attacks and strokes. ACE inhibitors, ARBs, β-blockers, calcium channel blockers, and antiarrhythmic agents manage hypertension, heart failure, and arrhythmias by improving blood flow and reducing cardiac workload. Diuretics alleviate symptoms of heart failure by removing excess fluid, while nitrates improve blood flow to the heart. Recent advancements include SGLT2 inhibitors, PCSK9 inhibitors, and neprilysin inhibitors, which offer additional benefits in managing heart failure and dyslipidemia. Fibrates, aldosterone antagonists, direct renin inhibitors, cholesterol absorption inhibitors, antisense oligonucleotides, ivabradine, and ranolazine provide further therapeutic options to address various aspects of CVDs. SGLT2 inhibitors are used for heart failure and glycemic control. PCSK9 inhibitors are used for lowering LDL cholesterol. Neprilysin inhibitors combined with ARBs are used for heart failure. Fibrates are used for lowering triglycerides and raising HDL cholesterol. Aldosterone antagonists are used for managing hypertension and heart failure. Direct renin inhibitors are used for hypertension. Cholesterol absorption inhibitors are used for enhancing lipid control. Antisense oligonucleotides targeting PCSK9 are used for LDL cholesterol reduction. Ivabradine is used for managing heart failure with reduced ejection fraction. Ranolazine is used for chronic angina management. Figure 14 represents the structures of various chemical inhibitors as discussed above and the Structures were retrieved from PubChem.com. Table 4 provides a comprehensive overview of the key characteristics of various medication classes used in cardiovascular disease management, emphasizing their primary mechanisms of action and specific clinical applications.

## 5. Surgical Interventions for Severe CVD Conditions

CVDs often require more than just medication and lifestyle changes, especially in severe cases. Various surgical interventions can provide significant relief, improve symptoms, and extend life expectancy for patients with advanced CVDs. These procedures target specific heart conditions, helping to restore normal function and prevent further complications. Some of the key surgeries used to treat different CVDs were discussed.

### 5.1. Coronary Artery Bypass Grafting (CABG)

Coronary artery bypass grafting (CABG) is a surgical procedure used to treat severe coronary artery disease (CAD). When one or more coronary arteries are blocked or severely narrowed, reducing blood flow to the heart muscle, CABG restores blood flow by bypassing the blocked arteries using grafts taken from other parts of the body, such as the saphenous vein from the leg or the internal mammary artery. This procedure alleviates angina, improves heart function, and reduces the risk of heart attacks [237,238]. CABG is particularly beneficial for patients with multi-vessel disease, left main coronary artery disease, or those who have not responded well to other treatments, like medication or percutaneous coronary interventions (PCIs). The surgery has been shown to improve survival rates, especially in high-risk patients, and enhances the overall quality of life by significantly reducing symptoms and the need for additional revascularization procedures. Post-surgery, patients typically undergo cardiac rehabilitation to optimize recovery and long-term heart health [239,240].

### 5.2. Percutaneous Coronary Intervention (PCI)

**Figure 15:**
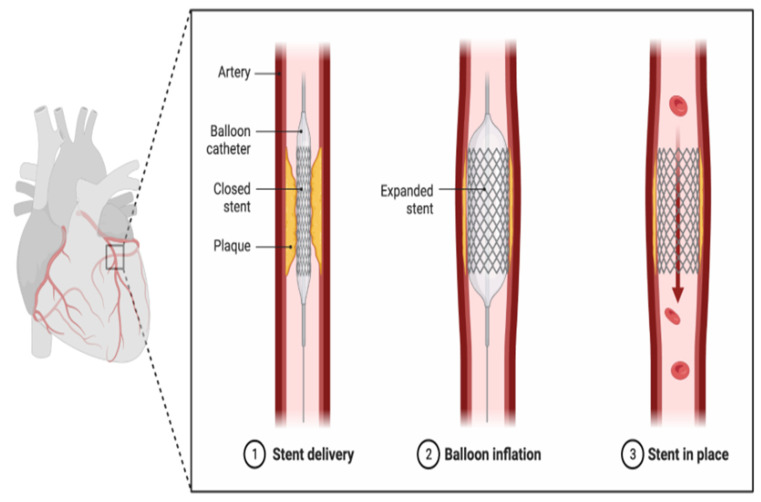
Percutaneous coronary intervention procedure steps involved stent delivery, balloon inflation, and stent in place. Adapted from “Percutaneous Coronary Intervention” by BioRender.com (https://app.biorender.com/biorender-templates, accessed on 15 July 2024).

PCI, commonly known as angioplasty with stent placement, is a minimally invasive procedure used to open blocked or narrowed coronary arteries. During PCI, a catheter with a balloon at its tip is inserted into the affected artery, and the balloon is inflated to widen the artery. A stent, a small wire mesh tube, is then placed to keep the artery open, improving blood flow and reducing symptoms of angina and the risk of heart attack (Figure 15). PCI is often preferred for its shorter recovery time compared to coronary artery bypass grafting (CABG), allowing patients to resume normal activities more quickly [241,242]. Advances in stent technology, including drug-eluting stents that release medication to prevent re-narrowing of the artery, have significantly improved long-term outcomes. PCI also plays a crucial role in the emergency treatment of heart attacks, enhancing survival rates and heart function recovery [243,244]. Primary PCI is a treatment of choice for treating myocardial infarction with ST segment elevation (STEMI). This procedure is particularly effective in restoring blood flow quickly, which is critical in limiting the extent of heart muscle damage during a heart attack. The swift intervention offered by PCI not only improves survival rates but also reduces the likelihood of severe complications, such as heart failure.

### 5.3. Heart Valve Repair or Replacement

Heart valve repair or replacement is performed to treat valvular heart diseases, such as stenosis (narrowing) or regurgitation (leakage) of the heart valves. Depending on the severity and type of valve disease, surgeons may repair the damaged valve or replace it with a mechanical or biological prosthetic valve. This surgery helps restore normal blood flow, reduce symptoms, like shortness of breath and fatigue, and prevent complications, such as HF and stroke [245,246]. Valve repair, when feasible, is often preferred over replacement as it preserves the patient’s native valve and avoids the need for lifelong anticoagulation therapy associated with mechanical valves. Advanced surgical techniques and minimally invasive approaches, such as transcatheter aortic valve replacement (TAVR), have expanded treatment options, especially for high-risk patients who are not candidates for traditional open-heart surgery [247,248]. The choice between mechanical and bioprosthetic valves depends on factors, like patient age, lifestyle, and medical history, with mechanical valves offering greater durability and biological valves providing the advantage of not requiring long-term blood thinners [249]. Post-surgery, patients typically experience significant improvements in quality of life, exercise capacity, and overall cardiovascular health, alongside a substantial reduction in mortality and morbidity rates associated with valvular heart diseases.

### 5.4. Pacemaker and/or Implantable Cardioverter–Defibrillator (ICD) Insertion

ICDs are devices used to treat arrhythmias, conditions where the heart beats too fast, too slow, or irregularly. A pacemaker helps regulate the heart rate by sending electrical impulses to stimulate heartbeats. An ICD monitors the heart’s rhythm and delivers electrical shocks to restore normal rhythm if a life-threatening arrhythmia is detected. These devices are implanted under the skin near the heart and connected to the heart with leads. The insertion of a pacemaker or ICD is typically performed under local anesthesia and sedation, making it a minimally invasive procedure. The leads are threaded through veins and positioned in the heart under X-ray guidance. Once in place, the device’s settings are programmed and tested to ensure proper function [250,251]. Regular follow-up appointments are essential to monitor the device’s performance and battery life. Patients may need to take precautions around certain electrical devices and undergo periodic device checks to ensure optimal functionality. These devices significantly improve the quality of life for patients with serious heart rhythm disorders, providing a sense of security and reducing the risk of sudden cardiac death.

### 5.5. Aortic Aneurysm Repair

Aortic aneurysm repair is a surgical procedure to treat aneurysms (bulging) in the aorta, the main artery carrying blood from the heart to the rest of the body. If an aneurysm becomes too large or is at risk of rupture, it can be repaired using open surgery or endovascular techniques [252,253]. Open surgery involves replacing the weakened section of the aorta with a synthetic graft. This procedure requires a large incision in the chest or abdomen to access the aorta. The aneurysmal section is removed and replaced with a durable graft that is sewn into place, ensuring the integrity of the aorta is restored. Recovery from open surgery can be extensive, requiring significant hospital stay and rehabilitation [253,254]. Endovascular repair, on the other hand, is a less invasive option that uses a stent graft placed inside the aneurysm through catheters inserted via the blood vessels, usually through small incisions in the groin. The stent graft reinforces the weakened section of the aorta, allowing blood to bypass the aneurysm, thus reducing the risk of rupture. This method typically results in shorter recovery times and fewer complications compared to open surgery [253,254,255]. Both procedures require careful preoperative planning and postoperative monitoring to ensure the repair is successful and to prevent complications. Regular follow-up imaging tests are necessary to monitor the graft and the aorta’s condition, ensuring long-term effectiveness and patient safety.

### 5.6. Heart Transplant

A heart transplant is a life-saving procedure for patients with end-stage HF who do not respond to other treatments. It involves replacing the diseased heart with a healthy donor heart. Although heart transplants can significantly improve survival and quality of life, they require lifelong immunosuppressive medications to prevent the rejection of the donor’s heart. This surgery is reserved for patients with severe heart conditions who have exhausted all other treatment options [256,257]. Heart transplant recipients must undergo a rigorous evaluation process to ensure they are suitable candidates for the procedure. The success of the transplant depends not only on the surgery itself but also on careful postoperative management and regular follow-ups. Potential complications include rejection, infection, and the side effects of immunosuppressive drugs, which can include an increased risk of certain cancers. Despite these challenges, many patients enjoy a significantly improved quality of life and increased longevity following a heart transplant. Advances in surgical techniques and postoperative care continue to improve outcomes for transplant recipients [258,259].

### 5.7. Left Ventricular Assist Device (LVAD) Implantation

LVADs are mechanical pumps that help the left ventricle, the heart’s main pumping chamber, circulate blood throughout the body. LVADs are used as a bridge to heart transplant or as a long-term solution for patients with advanced HF who are not candidates for transplant. By assisting the weakened heart, LVADs improve symptoms, enhance quality of life, and increase survival rates for patients with severe HF. The implantation of an LVAD requires major surgery and is followed by a period of intensive monitoring and adjustment. Patients with LVADs need to adhere to a strict medical regimen and regularly attend follow-up appointments to ensure the device is functioning properly. Complications can include bleeding, infection, and blood clots, which require vigilant management. Advances in LVAD technology have led to more durable and smaller devices, allowing patients greater mobility and a more normal lifestyle. The support provided by an LVAD can significantly delay the progression of HF and offer patients additional years of active life [260,261].

These surgical interventions, along with medications, play a critical role in managing and treating severe cardiovascular diseases, offering patients improved outcomes and quality of life.

## 6. Side Effects Associated with Various CVD Chemical Drugs

The treatment of cardiovascular diseases (CVDs) often involves the use of various chemical drugs, each with its potential side effects. Statins, commonly prescribed to lower cholesterol, can lead to muscle pain, liver damage, digestive problems, and increased blood sugar levels, which may cause diabetes [262]. Beta blockers can cause fatigue, cold extremities, weight gain, depression, and sexual dysfunction [263]. ACE inhibitors, used to manage hypertension, may result in a persistent dry cough, elevated blood potassium levels, low blood pressure, and kidney impairment. ARBs share similar side effects with ACE inhibitors but generally have a lower incidence of cough [264]. Calcium channel blockers can cause swelling in the lower extremities, constipation, dizziness, and an increased heart rate [265]. Diuretics, which help reduce fluid buildup, can lead to electrolyte imbalances, dehydration, and increased urination [266]. Antiplatelet agents, like aspirin, can cause gastrointestinal bleeding, ulcers, and allergic reactions. Anticoagulants, such as warfarin, have a risk of excessive bleeding, which can be life threatening [267]. Nitrates, used to relieve angina, may result in headaches, dizziness, and a rapid heartbeat [268]. Aldosterone antagonists can cause hyperkalemia, kidney dysfunction, and gynecomastia in men [269]. SGLT2 inhibitors, initially developed for diabetes but are now also used in HF, can lead to genital infections and urinary tract infections [270]. PCSK9 inhibitors, which lower LDL cholesterol, may cause flu-like symptoms, back pain, and allergic reactions at the injection site [271]. Fibrates, used to lower triglyceride levels and increase HDL cholesterol, can cause gastrointestinal disturbances, gallstones, liver enzyme abnormalities, and myopathy (muscle pain with creatine kinase elevations) [272]. Neprilysin inhibitors, combined with ARBs for HF management, can lead to hypotension, hyperkalemia, renal impairment, and angioedema [273]. Ivabradine, used for CHF, may cause bradycardia (slow heart rate), hypertension, atrial fibrillation, and visual disturbances, like luminous phenomena [274]. Ranolazine, used to treat chronic angina, can cause dizziness, headache, constipation, and nausea [275]. Due to the side effects associated with conventional cardiovascular drugs, there has been a growing interest in exploring herbal remedies for treating CVDs.

## 7. Herbal Remedies for Cardiovascular Diseases

As the population affected by CVDs continues to grow, the need for effective and safe treatment options becomes more critical. Herbal remedies, which originate from natural sources, are often regarded as safer alternatives to allopathic medications. Herbal medicines, while generally considered safe, can cause adverse effects if taken without proper monitoring. Prolonged use or overdose of these herbal medications can lead to significant side effects, including an increased risk of cardiovascular events. Standard pharmacovigilance techniques, as outlined by WHO guidelines, are essential for the regulation, usage, nomenclature, and public perception of herbal medicines. There is a pressing need for a broad and systematic approach to the use of herbal and traditional medicines. These herbal remedies contain a wide range of bioactive compounds, leading to a variety of cellular actions. For example, they can act as antioxidants, promote blood vessel relaxation, reduce inflammation, inhibit cell proliferation, and increase urine production. Herbal remedies may help prevent changes in smooth muscle cells, improve endothelial function, reduce platelet activation, lower levels of harmful lipid oxidation, decrease the production of ROS, and decrease the formation of atherosclerotic lesions by macrophages. Due to the array of molecular and cellular targets they impact, herbal remedies can effectively address various CVDs.

### Different Plant Extracts and Their Bioactive Compounds Used in the Prevention and Treatment of CVDs

*Digitalis purpurea*, known as foxglove, has been a cornerstone in the treatment of CVDs since its medicinal use was first documented by William Withering in 1785. The active components of this plant, primarily cardiac glycosides, such as digoxin and digitoxin, have been extensively utilized in managing various heart conditions due to their profound effects on cardiac function [276]. Digitalis glycosides work by inhibiting the sodium–potassium adenosine triphosphatase (Na^+^/K^+^-ATPase) pump. This inhibition leads to an increase in intracellular calcium concentrations, which enhances the contractility of the heart muscle. This positive inotropic effect is particularly beneficial in the treatment of HF, as it improves cardiac output and alleviates symptoms associated with reduced myocardial function [276,277]. These glycosides also increase vagal tone and slow conduction through the atrioventricular (AV) node. This results in a reduced heart rate, which is especially useful in managing atrial arrhythmias such as atrial fibrillation. By controlling the heart rate, digitalis helps in maintaining a more stable and manageable rhythm in patients with these conditions [278]. The improvement in cardiac output resulting from digitalis glycosides indirectly reduces fluid retention and symptoms of congestion. This diuretic effect helps alleviate edema and pulmonary congestion in HF patients, contributing to overall symptom relief and improved quality of life [279]. Cardiac glycosides can stabilize the heart’s electrical activity, making them effective in treating certain arrhythmias. Their ability to modulate electrical impulses is particularly beneficial in managing atrial fibrillation and atrial flutter, where they help restore and maintain normal heart rhythm. Digitalis compounds have been a standard of care for HFrEF and rate control in atrial fibrillation [276,277,278,279,280]. Despite newer treatments, digitalis glycosides remain important for specific clinical scenarios due to their ability to enhance cardiac contractility, control heart rate, manage fluid retention, and stabilize electrical impulses. However, their use requires careful monitoring due to the narrow therapeutic window, as excessive levels can lead to toxicity. Regular serum level checks are essential to balance therapeutic benefits with the risk of adverse effects, such as arrhythmias or gastrointestinal symptoms. 

*Allium sativum*, commonly known as garlic, effectively addresses hypertension, oxidative stress, inflammation, and hyperlipidemia. It lowers total cholesterol and LDL levels, inhibits vascular smooth muscle cell proliferation, manages atherosclerosis, and promotes endothelial nitric oxide synthase-mediated vasorelaxation. Furthermore, garlic has been shown to enhance immune function, improve blood circulation, and reduce the risk of CVD events. Allicin and other sulfur compounds in garlic have been shown to inhibit cholesterol synthesis in the liver and reduce overall lipid levels in the blood. This helps in lowering LDL cholesterol and total cholesterol levels. Garlic consumption has been linked to reductions in blood pressure, contributing to overall cardiovascular health [281,282,283]. Garlic has been found to reduce platelet aggregation, which can lower the risk of clot formation and improve cardiovascular function. Regular consumption of garlic is associated with a reduction in the severity of atherosclerosis. Studies suggest that garlic’s ability to lower cholesterol levels, reduce inflammation, and improve endothelial function plays a role in mitigating atherosclerotic changes [284,285]. 

*Astragalus membranaceus* is highly beneficial for ischemic-associated CVDs and improving cardiac function, primarily due to the presence of Astragaloside IV, a saponin. This compound enhances energy metabolism, inhibits free radicals, maintains cardiac function, reduces insulin resistance, and exhibits anti-obesity and hypolipidemic effects. Astragaloside IV has been shown to modulate the immune system, reduce inflammation, and protect against myocardial injury, further contributing to cardiovascular health. Studies also indicate its potential in promoting angiogenesis and improving microcirculation. Astragaloside IV mitigates myocardial ischemia/reperfusion injury in rats by inhibiting calcium-sensing receptor-mediated apoptotic signaling pathways [286,287]. Combination therapy of *A. membranaceus* with conventional therapy offers more advantages than alone in improving left ventricular remodeling and clinical efficacy in patients with HFrEF, without increasing the incidence of adverse reactions [288].

Hawthorn (*Crataegus* spp.) extracts have been traditionally used in treating CVDs due to their multifaceted mechanisms of action. Its berries, leaves, and flowers contain bioactive compounds, such as flavonoids and proanthocyanidins. These compounds help improve heart health by enhancing blood flow, reducing blood pressure, and preventing the formation of arterial plaques, which lead to atherosclerosis inhibition [289,290]. Berries are particularly rich in hyperoside, while leaves have higher levels of vitexin-2-rhamnoside and oligomeric procyanidins (OPCs), contributing to their therapeutic effects [289]. Hawthorn extracts also help in lowering blood pressure through vasorelaxation by stimulating the synthesis of nitric oxide (NO) and they have relaxant effects on smooth muscle, aiding in the reduction in vascular resistance [291]. The extracts exhibit anti-atherosclerotic effects through several mechanisms, including the downregulation of caspase-3 gene expression, the regulation of lipoprotein lipase expression, an increased excretion of bile acids via the upregulation of cholesterol 7α-hydroxylase activity, a reduced activity of intestinal Acyl CoA cholesterol acyltransferase, and the inhibition of thromboxane A2 (TXA2), reducing platelet aggregation and thrombus formation [292,293,294]. Additionally, hawthorn extracts have several direct effects on cardiac cells, such as the inhibition of 3′,5′-cyclic adenosine monophosphate phosphodiesterase, leading to increased coronary blood flow, enhanced relaxation velocity, slight positive inotropic effects, and a slight increase in heart rate, as well as chronotropic and antiarrhythmic actions, helping to stabilize heart rhythms and prevent arrhythmias [295,296]. The multifaceted actions of hawthorn extracts on the cardiovascular system make them a promising therapeutic option for managing and preventing CVDs.

*Crocus sativus*, commonly known as saffron, and its bioactive compounds, such as crocin and safranal, exhibit significant cardioprotective effects, making them valuable in the treatment of CVDs. These compounds mitigate ischemia–reperfusion injury (IRI) by reducing oxidative stress, inflammation, and apoptotic pathways. Crocin, in particular, demonstrates a dose-dependent inhibition of TNF-α and ICAM-1 expression, a reduction in lipid peroxidation (LPO), and an enhancement of antioxidant capacity in renal and myocardial IRI models. It also ameliorates left ventricular pressure, heart rate, and coronary flow while reducing ventricular tachycardia and fibrillation incidences [297,298]. Safranal supports cardiovascular health by decreasing infarct size, improving left ventricular function, and reducing TNF-α levels. It exhibits anti-apoptotic properties by upregulating Bcl-2 and downregulating Bax and caspase-3 expressions. Safranal’s ability to restore cardiac injury markers and augment antioxidant capacity underscores its therapeutic potential in managing myocardial injury. Additionally, safranal exerts anti-inflammatory properties and improves overall hemodynamic status. In the context of hyperlipidemia and atherosclerosis, saffron and crocin reduce oxidative stress and inhibit the oxidative modification of low-density lipoprotein (Ox-LDL), a critical factor in atherogenesis. These compounds enhance endothelial function by maintaining NO production, thus preventing endothelial dysfunction and subsequent atherosclerotic progression. In hyperlipidaemic models, saffron and crocin effectively lower serum triglycerides, total cholesterol, and LDL-C levels while increasing antioxidant enzyme activities, such as SOD and catalase. Additionally, crocin inhibits the formation of foam cells and smooth muscle cell proliferation, key processes in plaque formation and vascular occlusion [299,300,301,302]. Collectively, the bioactive compounds in *Crocus sativus* offer multifaceted benefits in treating CVDs by mitigating oxidative stress, inflammation, and apoptotic pathways, thus preserving cardiac function and structure. 

*Salvia miltiorrhiza*, commonly known as Danshen or Red Sage, is used to treat coronary heart disease, hypertension, atherosclerosis, MI, clogged arteries, and chest pain. Its efficacy is primarily attributed to its active components, which include salvianolic acids, tanshinones, and phenolics [303]. In hypertension, tanshinone IIA reduced pulmonary artery wall remodeling and right ventricular systolic pressure in hypoxia or monocrotaline-induced pulmonary hypertensive rats by modulating KV2.1 and KV1.5 expression and inhibiting basal intracellular Ca^2+^ concentration and store-operated Ca2^+^ entry through the downregulation of TRPC1 and TRPC6 expression [304]. Tanshinone IIA prevented cardiac fibrosis and left ventricular hypertrophy in renovascular hypertension rats by decreasing collagen volume content and modulating MMP-9, TIMP-1, and TIMP-2 expression [305]. Tanshinone IIA reduced monocyte chemotactic protein-1 (MCP-1), TGF-β1, TNF-α, and NF-κB expression, decreasing infarct sizes and collagen deposition in myocardial ischemia rats [306]. Danshensu or salvianolic acid A decreased blood pressure and inhibited arrhythmias in spontaneously hypertensive rats by increasing serum NO content and NO synthase activity [307]. Lithospermic acid B inhibited ACE plasma activities and attenuated Ang I-induced contraction in endothelium-intact aortic rings [308]. Magnesium tanshinoate B exhibited hypotensive effects on phenylephrine-induced elevated blood pressure in rats, while salvianolic acid B reduced average blood flow velocity in the liver in endothelin-induced portal hypertension mice [309,310]. Salvianolate reduces pro-inflammatory cytokines and increases regulatory T cells in atherosclerosis rats [311]. Danshensu improved coronary flow, heart rate, and left ventricular developed pressure in isolated rat hearts myocardial ischemia model, while Danshen increased HIF1α and VEGFA expression, improving cardiac function and angiogenesis in myocardial ischemia mice [312,313]. Cryptotanshinone inhibited NF-κB translocation and reduced pro-inflammatory cytokines in ischemic myocardial tissues and reduced oxidative stress and inflammation in HUVECs by inhibiting LOX-1, VCAM-1, ICAM-1, and E-selectin expression [314,315]. Tanshinone IIA inhibited ET-1 release and stimulated NO production via eNOS activation and ATF-3 expression HUVECs [316]. Tanshinones inhibited vascular smooth muscle cell (VSMC) proliferation by decreasing ERK1/2 signaling and cyclin D expression while increasing p21waf1/cip1 expression [317]. Danshensu and salvianolic acid B inhibited TNF-α induced hyperpermeability by reducing VEGF expression in HUVECs [318]. *S. miltiorrhiza* bioactive compounds have shown effectiveness in treating various cardiovascular risk conditions. Further research will help transition from laboratory studies to clinical trials and regular commercial use.

*Centella asiatica* is renowned for its triterpenoid saponins, including asiatic acid, asiaticoside, and madecassoside, which contribute to its cardioprotective effects [319]. The aqueous extract of *C. asiatica* is effective in attenuating elevated serum myocardial marker enzymes in cardiomyopathy rats, indicating a reduction in cardiac damage. The total triterpene fraction from *C. asiatica* enhances microcirculation and capillary permeability in patients with venous hypertension [320,321]. Asiatic acid exhibits multiple benefits: it prevents cardiomyocyte hypertrophy induced by TGF-β1 and IL-1β, reduces angiotensin II-induced hypertrophy and collagen accumulation, and alleviates cardiac hypertrophy and fibrosis in pressure overload-induced mice [322,323,324]. Additionally, asiatic acid improves cardiac function and reduces left ventricular remodeling following coronary artery ligation-induced myocardial infarction [325]. It also enhances hemodynamics in hypertensive and metabolic syndrome rats by reducing blood pressure, improving NO bioavailability, and addressing vascular dysfunction [326,327,328]. Asiaticoside contributes by lowering systemic blood pressure and improving NO and cGMP production in hypoxia-induced pulmonary hypertensive rats [329]. Madecassoside protects against ischemia–reperfusion injury-induced myocardial infarction, reduces plasma TNF-α levels, and prevents hypotension and tachycardia in LPS-induced rats [330,331]. 3,5-di-O-caffeoylquinic acid exhibits antithrombotic properties and inhibits platelet reactivity [332]. Collectively, these compounds from *C. asiatica* offer a comprehensive range of cardioprotective benefits through mechanisms that include reducing hypertrophy, fibrosis, ischemic damage, and inflammation.

Ginseng (*Panax* sp.) has been extensively studied for its cardioprotective properties, which are largely attributed to its active triterpene saponins, which are known as ginsenosides. Ginsenosides facilitate vasorelaxation across various vessels, including aortas, coronary arteries, and cerebral arteries [333,334]. They enhance arterial function by increasing the expression of endothelial NO synthase (eNOS) and promoting NO production. Specifically, ginsenoside Rg3 activates eNOS, leading to NO-dependent vasorelaxation that improves vascular tone [335]. Additionally, Ginseng G115 extract has been shown to inhibit ACE activity, further contributing to its antihypertensive effects [336]. Beyond its blood pressure-lowering capabilities, ginseng exhibits significant antioxidant, anti-inflammatory, and anti-hyperlipidemic properties, which are critical in mitigating the progression of atherosclerosis and other cardiovascular conditions. Ginsenosides inhibit the activation of key inflammatory pathways, including activator protein-1 (AP-1) and NF-κB. This inhibition results in decreased expression of pro-inflammatory cytokines such as COX-2, IL-6, IL-1β, and TNF-α [337]. Dietary supplementation of ginseng has been shown to lower blood cholesterol levels and reduce the formation of atherosclerotic lesions. Furthermore, ginseng demonstrates potent antithrombotic effects, primarily through antiplatelet rather than anticoagulation activity. Dihydro-ginsenoside Rg3, for instance, inhibits platelet aggregation by modulating intracellular signals, such as cAMP and ERK2, making ginseng beneficial for individuals at high risk of thrombosis and CVDs [338].

Roselle (*Hibiscus sabdariffa*) has been extensively studied for its beneficial effects on cardiovascular health, with various parts of the plant demonstrating significant cardioprotective properties. The calyx, rich in polyphenols, has been shown to reduce serum TG levels and systolic blood pressure in patients with metabolic syndrome [339]. Roselle calyx extract in a jelly drink form decreases levels of LDL-C, TNFα, and MDA while increasing GSH levels, contributing to improved lipid profiles and reduced oxidative stress in individuals with dyslipidemia [340]. A standardized herbal medicinal product containing roselle has effectively reduced blood pressure, lowered triglycerides, and decreased mean serum renin and ACE activity in patients with grade 1 essential hypertension [341]. Roselle sour tea has been beneficial for diabetic patients with mild hypertension by decreasing systolic blood pressure and mean pulse pressure. Additionally, roselle infusion has been found to lower pulse pressure, heart rate, and ventricular hypertrophy in subjects with moderate essential hypertension [342,343]. Roselle flower tea and dried flower infusion have demonstrated the ability to lower systolic and diastolic blood pressure in patients with hypertension [344]. Moreover, roselle calyx tea has been effective in reducing postprandial systolic and diastolic blood pressure, serum glucose, plasma insulin, serum TGs, and CRP levels, while improving antioxidant response [345]. Overall, various parts of the roselle plant contribute significantly to cardiovascular health by improving blood pressure, lipid profiles, and antioxidant status.

*Ginkgo biloba* extract (GBE) provides substantial cardioprotective effects through its active compounds, ginkgolides, and bilobalide. GBE exhibits vasodilatory and antihypertensive properties, as well as ACE inhibitory activities. GBE promotes endothelial health, activates cholinergic pathways, inhibits endothelial activation and adhesion, and has lipid-lowering effects, all contributing to its cardioprotective benefits [346,347]. GBE reduces adipogenesis and enhances lipolysis via ginkgolide C, which modulates the Sirt1/AMPK pathway, decreases acetyl-CoA carboxylase activity, and boosts lipolysis by stimulating adipose triglyceride lipase and hormone-sensitive lipase. Additionally, Ginkgolide C inhibits lipid accumulation by lowering the expression of PPAR transcription factors associated with adipogenesis [348]. GBE also combats endothelial dysfunction and reduces vascular inflammation. Ginkgolide B, a major component, inhibits the production of MCP-1, intercellular adhesion molecule-1 (ICAM-1), and vascular cell adhesion molecule-1 (VCAM-1) in ox-LDL-induced endothelial cells, while also decreasing inflammatory cytokine expression in macrophages [349]. GBE diminishes the production of MMP-1, an enzyme involved in atherosclerotic plaque rupture, in ox-LDL-induced coronary smooth muscle cells [350]. In terms of vascular function, GBE enhances endothelial health, performs ACE inhibitory activities, and activates cholinergic pathways. It exhibits vasodilatory and antihypertensive effects, lowers serum lipid levels, and reduces the proliferation and morphological changes of VSMCs induced by LPS. GBE also regulates vascular inflammation by decreasing the activity of NADPH oxidase (NOX) and reducing MAPK phosphorylation, which suppresses toll-like receptor 4 expression in smooth muscle cells [351].

*Camellia sinensis*, commonly known as green tea, is highly regarded for its cardiovascular protective effects, primarily due to its rich content of polyphenols and flavonoids. Key compounds include green tea catechins (GTCs) such as epigallocatechin gallate (EGCG), catechin, epicatechin, gallocatechin, gallocatechin gallate, epigallocatechin, and epicatechin gallate [352]. Many studies have shown that GTCs exert cardioprotective effects through multiple mechanisms. These include inhibiting oxidative processes, reducing vascular inflammation, and preventing thrombogenesis [353,354,355]. GTCs such as EGCG have demonstrated beneficial effects, such as improved endothelial function and reduced hypertension, by enhancing NO production and vasodilation [356]. EGCG can reverse endothelial impairment and enhance brachial artery flow-mediated dilation in individuals with CAD [357]. Additionally, GTCs exhibit antihypertensive properties by elevating plasma NO levels, which inhibit pro-inflammatory cytokines and platelet aggregation while improving endothelial function [352]. They also show anti-inflammatory effects by suppressing inflammatory cytokines and adhesion molecules and may exert vasodilatory effects by inhibiting the expression of eNOS and endothelin-1 [358,359]. Thus, bioactive compounds in *Camellia sinensis*, particularly GTCs, offer significant benefits by reducing oxidative stress, alleviating inflammation, protecting endothelial function, improving lipid profiles, and enhancing overall vascular health.

Terminalia extracts exhibit a wide range of cardioprotective effects, including impacts on cardiac hemodynamics, coronary flow, and blood pressure. The bark of Terminalia is noted for its diuretic, inotropic, and chronotropic properties. It has been shown to enhance coronary flow and increase the force of cardiac muscle contraction, which is beneficial in treating heart conditions. Terminalia extracts can lower blood pressure, with effects mediated through various mechanisms, including potential cholinergic pathways [360,361,362]. Terminalia extracts augment endogenous antioxidants in the heart, offering protection against oxidative stress and IRI [363]. Arjunolic acid from the bark extract of Terminalia exhibited effective cardioprotective activity by boosting the endogenous antioxidant system. Arjunolic acid has been shown to effectively maintain levels of key antioxidants and protective compounds in the heart. It prevents decreases in SOD, CAT, GPx, ceruloplasmin, α-tocopherol, GSSH, and ascorbic acid. Additionally, it mitigates increases in LPO and MPO, thereby supporting the heart’s defense against oxidative stress [364,365]. The extracts show promise in protecting against myocardial damage induced by harmful agents and in ameliorating conditions, like congestive HF and myocardial ischemia [364]. *T. arjuna* extracts protect the heart from myocardial alterations and reduce myocardial fibrosis and oxidative stress caused by prolonged β-adrenoceptor activation [366]. Terminalia arjuna extracts exhibit inotropic properties and possess anti-ischemic, antiplatelet, hypolipidemic, antiatherogenic, antihypertrophic, and blood pressure-lowering effects [367]. Terminalia extracts have been used to alleviate symptoms of angina, MI, and other CVDs. The extracts also offer significant benefits in conditions such as ischemic mitral regurgitation and rheumatic heart disease [368,369,370]. 

*Tinospora cordifolia* extracts have shown significant protection against isoprenaline-induced cardiotoxicity in diabetic rats. The cardioprotective effects include a reduction in infarct size and serum LPO levels, attributed to the extract’s radical scavenging activity, the protection of the Mg^2+^-dependent Ca^2+^-ATPase enzyme, the inhibition of sarcolemmal Na^+^-K^+^-ATPase activity, and Ca^2+^ channel blocking activity. The alcoholic extract of *T. cordifolia* has also demonstrated significant cardioprotective activity in ischemia–reperfusion-induced myocardial infarction, effectively reducing infarct size and oxidative stress markers. In another study, the extract exhibited protective effects against calcium chloride-induced cardiac arrhythmia, indicating its broader potential in managing various cardiac conditions [371,372,373]. Furthermore, the stem extract of *T. cordifolia* has been found to normalize lipid metabolism alterations in diabetic rats, indirectly benefiting heart health by reducing serum and tissue total cholesterol, phospholipids, and free fatty acids. In cases of cadmium-induced cardiotoxicity, the methanolic stem extract of *T. cordifolia* restored enzyme levels and antioxidant statuses, showcasing its efficacy as a cardioprotective agent. Additionally, the root extract has been shown to attenuate isoprenaline-induced MI, suggesting its ability to fortify myocardial membranes and mitigate cardiac damage [374,375,376]. 

*Nerium oleander* Linn (NOL), an evergreen shrub, is recognized for its extensive range of bioactivities with significant implications for cardiovascular health. NOL extract counteracts myocardial toxicity induced by isoproterenol. This is evidenced by its ability to normalize elevated levels of key cardiac enzymes and reduce LPO, which is crucial for preventing oxidative damage. The extract helps maintain antioxidants, such as SOD and GSH, thereby enhancing the heart’s resilience to oxidative stress. The role of NOL in regulating oxidative stress through its effects on hypoxia-inducible factors and antioxidant enzymes underscores its potential therapeutic applications [377,378].

Carrot roots (*Daucus carota*) are linked to reduced cardiovascular risk and metabolic disorders due to their rich content of bioactive compounds like carotenoids, vitamins, polyphenols, fiber, and minerals. Carrot antioxidants, such as carotenoids and polyphenols, effectively scavenge ROS, thereby reducing oxidative stress and lowering CVD risk. Purple carrot juice in the rat models of metabolic syndrome improved cardiovascular and hepatic structure and function, along with associated metabolic parameters, like abdominal fat deposition and plasma lipid profiles [379,380,381].

Amaranthus species, including *A. viridis*, *A. caudatus*, *A. spinosus*, *A. cruentus*, and *A. hypochondriacus*, have shown promising cardioprotective effects. Studies demonstrate that *A. viridis* methanolic extract and its isolated compound kaempferol significantly protect against isoproterenol-induced cardiotoxicity in rats. These extracts reduce cholesterol and TG levels while increasing HDL. They also enhance the activities of key antioxidant enzymes and reduce the production of pro-inflammatory cytokines, thereby mitigating oxidative stress and inflammation [382]. The ethanolic extract of *A. caudatus* exhibits anti-atherosclerotic effects, significantly reducing cholesterol, LDL, TGs, oxidized LDL, and inflammatory markers in high cholesterol-fed rabbits. Additionally, hydroalcoholic extracts of *A. caudatus* show similar benefits, further highlighting their cardiovascular protective potential [383,384]. Leaf extracts of *A. spinosus*, both alone and in combination with vitamin C, have been shown to reverse adverse alterations caused by a high-fat diet in rats. These extracts reduce LPO and enhance antioxidant enzyme levels, supporting cardiovascular health [385]. Novel peptides derived from *A. cruentus* grains have been identified as potent inhibitors of HMG-CoA reductase, a key enzyme in cholesterol biosynthesis. This hypocholesterolemic effect is beneficial for cardiovascular health [386]. Unprocessed and extruded hydrolysates from *A. hypochondriacus* demonstrate anti-inflammatory effects by reducing the expression of interleukins and molecular markers involved in atherosclerosis in human macrophages [387].

Resveratrol, a natural polyphenol found in 70 different plant species, grapes, peanuts, and red wine, is renowned for its cardiovascular protective effects. Resveratrol, a polyphenol found in red wine and certain berries, plays a crucial role in cardiovascular health through its antioxidant properties. Resveratrol induces cellular antioxidants in cardiomyocytes, providing protection against oxidative and electrophilic injury. Resveratrol reduces endothelial oxidative stress by modulating the gene expression of key antioxidant enzymes like SOD1, GPx1, and Nox4. Resveratrol prevents superoxide-dependent inflammatory responses, further underscoring its protective effects in cardiovascular conditions [388,389,390]. Resveratrol significantly improves endothelial function in the aortas of diabetic mice by enhancing acetylcholine-induced vasorelaxation. This vascular protective effect is primarily mediated through the inhibition of the TNFα-induced activation of NADPH oxidase, which, in turn, reduces H_2_O_2_ formation and preserves eNOS function. Resveratrol effectively attenuates TNFα expression, further contributing to its protective role in vascular health [391]. The administration of resveratrol to apo E-deficient mice mitigated the progression of atherosclerotic lesions and peri-arterial fat deposition, accompanied by a decreased expression of ICAM-1 and VCAM-1 in affected blood vessels [392]. Resveratrol hinders VEGF-induced angiogenesis by disrupting the VEGF-induced tyrosine phosphorylation of vascular cadherin and its partner protein β-catenin. This inhibition involves blocking ROS-dependent activation of Src kinase and subsequent tyrosine phosphorylation of vascular endothelial cadherin. VEGF triggers O2- production via Nox activation. Resveratrol potentially inhibits Nox in this angiogenesis model, as indicated by studies using specific antisense peptides to prevent VEGF-induced endothelial cell signaling and angiogenesis [393,394,395]. 

Quercetin, a widely prevalent flavonoid found in numerous plants, boasts significant medicinal benefits for CVD. It exhibits powerful antioxidant properties, reduces platelet aggregation, and mitigates myocardial fibrosis. Additionally, quercetin enhances ventricular remodeling and cardiac function, protects vascular endothelium, and combats arrhythmia and heart failure. It also prevents IRI and helps regulate blood pressure [396].

Total flavonoids from *Anchusa italica* (TFAI) offer a powerful protective effect against chronic MI, improving cardiac function and remodeling. These benefits are partially due to TFAI’s anti-inflammatory properties and the inhibition of the PI3K/Akt/mTOR signaling pathway. *A. italica* Retz. contains rutin, hesperidin, quercetin, kaempferol, and naringenin, which provide anti-inflammatory, antioxidant, and anticoagulant effects, making it effective for ischemic heart disease and myocardial infarction [397,398].

Luteolin demonstrates robust cardioprotective effects across various CVDs such as CAD, HF, and atherosclerosis. It significantly reduces myocardial infarct size, both in ischemia/reperfusion-induced and spontaneous infarctions and decreases LDH release. Luteolin also lowers the incidence of arrhythmias and apoptotic cell death, as evidenced by a favorable shift in the Bax to Bcl-2 ratio. Moreover, it attenuates inflammation markers (IL-6, TNF-a, IL-1a), serum cardiac enzymes, and apoptosis pathways involving AKT and ERK signaling. These effects collectively contribute to reduced mortality rates and improved outcomes in myocardial infarction scenarios. Luteolin reduced the excessive growth of VSMCs, which is a common feature in hypertensive remodeling. Luteolin suppressed VSMC migration, and the migration of VSMCs contributes to vessel narrowing and remodeling. By inhibiting migration, luteolin may help maintain vessel integrity. Luteolin could be a potential therapeutic agent for managing hypertensive vascular remodeling [399,400,401,402].

Naringenin, a dietary flavanone, primarily lowers blood pressure, regulates nitric oxide levels, and safeguards against endothelial dysfunction. Hesperidin, a dietary flavanone, has antihypertensive properties. Both compounds can slow the development of atheroma plaques due to their anti-inflammatory actions. Additionally, the antioxidant properties of hesperetin enhance nitric oxide production and decrease calcium ion concentration, resulting in smooth muscle relaxation in blood vessels [403,404,405]. Isoflavones, such as Daidzein and Genistein, and flavonols, such as kaempferol, exhibit effective antihypertensive and antiatherosclerosis effects through different mechanisms [398].

Caffeic acid, found in Maqui, exhibits antiplatelet properties by inhibiting platelet aggregation stimulated by ADP and thrombin. It also increases cAMP-dependent protein phosphorylation in collagen–platelet interactions and decreases the production of thrombogenic molecules in human platelets. Dihydrocaffeic acid and dihydroferulic acid, which are derivatives of caffeic and ferulic acids, have been identified as potent antiplatelet agents. They effectively reduce ADP-stimulated platelet activation, measured by P-selectin expression and fibrinogen binding [406,407]. 

**Figure 16:**
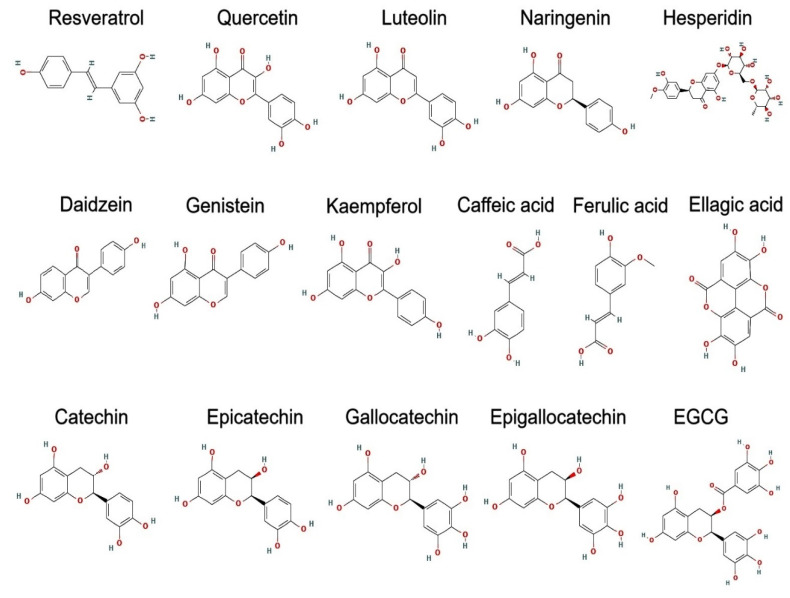
The structures of flavonoids and polyphenols showing significant cardioprotective effects. Structures were retrieved from the PubChem database.

Ellagic acid inhibits platelet activation induced by ADP and collagen, showcasing its antiplatelet properties. Ferulic acid demonstrates potential by activating cAMP and cGMP signaling. It inhibits platelet aggregation induced by various agonists and reduces intracellular Ca^2+^ mobilization and TxA2 production. Ferulic acid also increases cAMP and cGMP levels while decreasing phospho-MAPK and PDE in platelets. It has an antithrombotic effect in vivo and decreases AKT phosphorylation in thrombin-stimulated platelet activation. These phenolic acids, through their antiplatelet and antithrombotic activities, contribute significantly to cardiovascular protection [408,409,410]. Overall, different polyphenols and flavonoids from different plants exhibited effective cardioprotective effects through the various mechanisms clearly discussed, and the structures of these flavonoids and polyphenols were retrieved from PubChem and represented (Figure 16).

Saponins demonstrate various cardioprotective effects through their antioxidant, anti-inflammatory, and vasodilatory properties. Steroidal saponins from *Allium chinensis* reduce oxidative injury markers, while saponins such as glycyrrhizic acid (from *Glycyrrhiza glabra*), asperosaponin VI (from *Dipsacus asper* Wall), astragaloside IV (from *Astragalus membranaceus*), elatoside C (from *Aralia elata*), tribulosin (from *Tribulus terrestris*), platycodin D (from roots of *Platycodon grandiflorum*), and protodioscin and trillin (from rhizomes of *Dioscorea nipponica*) enhance antioxidant enzyme activity, protecting against ROS-induced cardiac damage [411]. Glycyrrhizic acid exhibits cardioprotective effects against isoproterenol-induced AMI in rats by significantly increasing SOD and GSH levels while reducing myocardial lipid hydroperoxide and 8-isoprostane levels. GA treatment also mitigates severe myocardial necrosis, nuclear pyknosis, and hypertrophy, highlighting its potent antioxidant properties [412]. Asperosaponin VI (ASA VI) exhibits significant cardioprotective effects in chronic MI in rats. It improves cardiac function, as evidenced by enhanced left ventricular systolic pressure (LVSP), reduced cardiac fibrosis, and decreased infarct size. ASA VI also lowers levels of pro-inflammatory cytokines (TNF-α and IL-6) while increasing anti-inflammatory IL-10. It boosts antioxidant defenses by elevating catalase, SOD, and GSH-Px activities and reduces oxidative stress markers, such as MDA. These effects contribute to its protective role against myocardial ischemia [413]. Elatoside C protects against hypoxia/reoxygenation (H/R)-induced injury in H9c2 cardiomyocytes by enhancing cell viability, preserving mitochondrial function, and reducing apoptosis. This cardioprotection is achieved through the inhibition of ER stress-associated apoptosis markers and the activation of STAT3 signaling, which helps mitigate mitochondrial ROS and apoptosis [414]. Tribulosin demonstrates cardioprotective effects by significantly reducing infarct size, MDA, AST, CK, and LDH levels while increasing SOD activity in cardiac ischemia/reperfusion injury isolated rat hearts. It lowers myocardial apoptosis rates in a concentration-dependent manner, enhances Bcl-2 and PKCɛ expression, and decreases Bax and caspase-3 expression [415]. Platycodin D exhibits cardioprotective effects by increasing NO levels and reducing MDA levels in endothelial cells. It also decreases monocyte adhesion and lowers the expression of adhesion molecules VCAM-1 and ICAM-1 induced by oxLDL, suggesting its potential as an anti-atherosclerotic agent [416]. Clematichinenoside, a triterpenoid saponin derived from Clematis chinensis, offers cardioprotection in ischemia/reperfusion injury through its antioxidant properties, which restores the balance between inducible NO synthase and endothelial NO synthase. It significantly reduces infarct size and enhances hemodynamic parameters. Additionally, Clematichinenoside lowers LDL, creatine kinase, and MDA levels while boosting SOD activity, indicating its effectiveness through antioxidant mechanisms [417]. Sasanquasaponin (SQS), a component of Camellia oleifera, offers cardioprotection against ischemia–reperfusion (I/R) injury by improving heart function and reducing arrhythmias. SQS mitigates oxidative damage by increasing SOD and GSH-Px activity and decreasing MDA and ROS levels. The cardioprotective effects of SQS are likely due to its activation of the bradykinin-NO system, which inhibits ROS generation in the heart [418]. Ophiopogonin D (OP-D) from tubers Ophiopogon japonicus provides cardioprotection by inhibiting oxidative stress, inflammation, and apoptosis in HUVECs. It reduces H_2_O_2_-induced lipid peroxidation and protein carbonylation, attenuates mitochondrial ROS generation, and decreases cell apoptosis. OP-D restores total antioxidative capacity, suppresses inflammatory cytokine release, and inhibits catalase, HO-1, and caspase activities while blocking NF-κB and ERK signaling pathways [419]. *Acanthopanax senticoside* B from *Acanthopanax senticosus* protects cardiomyocytes from oxidative damage by boosting catalase, GSH-Px, SOD, and GSSH levels [420]. Fistulosaponin A, a steroidal saponin from *Allium fistulosum,* showed anti-ischemic effects [421]. Saponins such as dioscin, diosgenin, and protodioscin from Dioscorea nipponica improve lipid profiles and exhibit anti-hyperlipidemic activity [422]. Various other saponins such as triterpenoid saponins from *Clematis tangutica*, Dioscin from Dioscorea species, reinioside C from *Polygala fallax*, Bacoside A3 and bacopaside II from *Bacopa monnieri*, Cistocardin from *Tribulus cistoides*, and β-Ascin from *Aesculus hippocastanum* improve endothelial dysfunction, while ginsenosides Rg1 and Rb1, tanshinone IIA, astragaloside IV, and salvianolic acid B show potential for cardiac regeneration [411].

Various plant alkaloids such as ajmalicine, ajmaline, reserpine, serpentine, and yohimbine from *Ravolfia serpentina* are notable for their diverse cardioprotective effects. The alkaloids Berberine from *Stephania japonica* and *Stephania cepharantha*, dehydroevodiamine from *Evodiae rutaecarpa*, dauricine and daurisoline from *Menispermum duricum*, rhynchophylline and hirsutine from *Uncaria rhynchophylla,* Fangchinoline and tetrandrine from the Stephania genus, and α-tomatine and tomatidine from tomato exhibit a range of mechanisms, including vasodilation to lower blood pressure and the prolongation of A-H and H-V intervals to extend ventricular action potentials. They also modulate calcium influx through voltage-sensitive channels and display negative inotropic effects by interacting with sodium channels. Plant-derived alkaloids offer substantial benefits, underscoring their importance in cardiovascular therapy [423]. 

## 8. Future Directions

Based on the comprehensive insights presented in this review, several promising avenues for future research and innovation in CVD management have been identified. These directions aim to enhance therapeutic efficacy, personalize treatment approaches, and integrate novel diagnostic and therapeutic technologies. Key future directions include the following.

Global Health Initiatives. Addressing CVDs in low- and middle-income countries requires tailored strategies that consider local health infrastructure, cultural practices, and economic constraints. Global health initiatives should focus on equitable access to diagnostics, treatments, and preventive measures.

Lifestyle Management. The importance of lifestyle management is emphasized, including regular exercise, walking, healthy eating habits, avoiding tobacco, and limiting alcohol consumption. Future research should focus on the impact of these lifestyle changes on CVD prevention and management, as well as strategies for promoting sustainable healthy behaviors.

Personalized Medicine and Genomic Profiling. Advances in genomics and personalized medicine offer the potential to tailor treatments to individual genetic profiles. Research should focus on identifying genetic markers associated with CVDs and developing targeted therapies that address specific genetic predispositions.

Development of Novel Biomarkers. The identification and validation of new cardiac biomarkers could improve early diagnosis and prognosis of CVDs. Emphasis should be placed on discovering biomarkers that can predict treatment response and disease progression.

Innovations in Imaging Techniques. Enhancing the resolution and accuracy of molecular imaging techniques, such as PET, MRI, and SPECT, can provide deeper insights into myocardial perfusion, plaque characterization, and overall cardiac function. Research should aim to develop less invasive, more precise imaging modalities.

Exploring New Pharmacological Targets. Identifying novel pharmacological targets and developing new classes of drugs could provide alternative treatment options for CVDs, particularly for patients who are unresponsive to existing therapies or suffer from severe side effects.

Integration of Herbal Remedies with Conventional Therapies. Further investigation into the synergistic effects of combining herbal remedies with conventional pharmacological treatments could enhance therapeutic efficacy while potentially reducing adverse side effects. Rigorous clinical trials are necessary to validate the safety and effectiveness of these integrative approaches. Longitudinal studies are needed to assess the long-term cardiovascular benefits and potential risks associated with the use of herbal remedies. These studies should also evaluate the impact of diet, lifestyle, and environmental factors on the effectiveness of herbal treatments.

Telemedicine and Digital Health Tools. Leveraging telemedicine and digital health technologies can improve patient monitoring, adherence to treatment, and overall management of CVDs. Future research should explore the integration of these tools into routine cardiovascular care.

By exploring these future directions, the medical community can enhance the understanding, diagnosis, and treatment of cardiovascular diseases, thereby improving patient outcomes and reducing the global burden of CVDs.

**Table 4:** A summary of the key characteristics of each medication class, highlighting their primary mechanisms and clinical applications in the management of cardiovascular diseases.

Medication Class	Examples	Primary Mechanism	Clinical Application	References
Statins	Atorvastatin Fluvastatin	Inhibit HMG-CoA reductase, reducing cholesterol production	Lowers LDL cholesterol, reduces the risk of heart attacks and strokes, and stabilizes atherosclerotic plaques	[158,159,160,161]
Anticoagulants	Warfarin, Rivaroxaban, Apixaban	Inhibit the coagulation cascade, preventing blood clots	Prevents and treats conditions like atrial fibrillation, DVT, PE, and stroke	[162,163,164,165,166,167]
Antiplatelet Agents	Aspirin, Clopidogrel, Ticagrelor	Prevent platelet aggregation, reducing blood clot formation	Manages acute coronary syndrome (ACS), prevents recurrent cardiovascular events, and maintains stent patency	[168,169,170,171,172]
ACE Inhibitors	Lisinopril, Enalapril	Inhibit conversion of angiotensin I to angiotensin II, leading to vasodilation and reduced blood pressure	Lowers blood pressure, treats heart failure (HF), and prevents heart and vessel remodeling	[173,174,175,176]
ARBs (Angiotensin II Receptor Blockers)	Losartan, Valsartan	Block angiotensin II receptors, preventing vasoconstriction	Treats hypertension, heart failure, and chronic kidney disease	[177,178,179,180]
Antiarrhythmic Agents—Class I (Sodium Channel Blockers)	Quinidine, Procainamide, Flecainide	Block sodium channels, stabilizing cardiac cell membranes and reducing conduction speed	Treats atrial fibrillation and ventricular arrhythmias Reduce the frequency of arrhythmic events and prevent the reoccurrence of arrhythmias	[181,182,183,184,185,186]
Antiarrhythmic Agents—Class II (β-blockers)	Metoprolol Propanolol	Block effects of adrenaline on the heart, reducing heart rate and blood pressure	Treats hypertension, angina, and arrhythmias, improves survival rates post-MI, prevents sudden cardiac death, manages HF symptoms	[187,188,189,190]
Antiarrhythmic Agents—Class III (Potassium Channel Blockers)	Amiodarone Sotalol	Block potassium channels, prolonging the repolarization phase of the cardiac action potential	Treats atrial fibrillation and ventricular tachycardia Effective for a broad range of arrhythmias	[191,192,193,194,195]
Antiarrhythmic Agents—Class IV (Calcium Channel Blockers)	Amlodipine, Verapamil, Diltiazem	Inhibit calcium ions from entering cardiac and smooth muscle cells, relaxing and widening blood vessels	Hypertension, angina, arrhythmias Improve myocardial oxygen delivery and control the ventricular rate in AF patients	[196,197,198,199,200,201]
Fibrates	Fenofibrate, Gemfibrozil	Activate PPARs, regulating lipid metabolism, lowering triglycerides, and increasing HDL cholesterol	Treats dyslipidemia, particularly in patients with high triglycerides and low HDL cholesterol	[202,203,204,205]
Neprilysin Inhibitors	Entresto (Sacubitril-Valsartan)	Inhibit neprilysin, enhancing natriuretic peptides and reducing angiotensin II effects	Synergistic effect of ARBs reduces hospitalization and mortality in HFrEF patients	[206,207,208,209,210]
Aldosterone Antagonists	Spironolactone, Eplerenone	Block aldosterone effects, promoting sodium and water excretion	Treats heart failure and resistant hypertension, reduces myocardial fibrosis and ventricular remodeling, improves long-term cardiac function	[211,212,213,214]
Diuretics	Furosemide, Hydrochlorothiazide	Increase urine production, reducing blood volume and pressure	Treats hypertension, heart failure, and symptoms like edema and pulmonary congestion	[215,216]
Nitrates	Nitroglycerin, Isosorbide Dinitrate	Dilate blood vessels by releasing nitric oxide, improving blood flow to the heart and relieving angina	Manages angina and heart failure by reducing heart workload and increasing oxygen delivery	[217,218]
Sodium–Glucose Cotransporter-2 Inhibitors (SGLT2)	Empagliflozin, Dapagliflozin	Inhibit SGLT2 in the kidneys, reducing glucose reabsorption, promoting diuresis, and alleviating heart failure	Diabetes, heart failure. Reduce HF hospitalizations, cardiovascular death, and all-cause mortality	[219,220,221]
PCSK9 Inhibitors	Alirocumab, Evolocumab	Inhibit PCSK9, increasing LDL receptor availability, lowering LDL cholesterol levels.	Lowers LDL cholesterol and reduces cardiovascular events in patients not adequately controlled by statins	[222,223,224,225,226]
Direct Renin Inhibitors	Aliskiren	Inhibit renin, preventing the formation of angiotensin I, reducing blood pressure and cardiac stress	Manages hypertension, particularly resistant cases	[227,228]
Cholesterol Absorption Inhibitors	Ezetimibe	Block cholesterol absorption in the intestines	Lowers LDL cholesterol and is often used in combination with statins	[229,230]
Antisense Oligonucleotides	Inclisiran	Reduces PCSK9 levels through RNA-based mechanisms	Enhances LDL cholesterol clearance, offering cardiovascular benefits	[231,232]
Ivabradine	Ivabradine	Selectively lowers heart rate by inhibiting the If current in the sinoatrial node	Manages chronic heart failure, particularly in patients symptomatic despite beta-blocker therapy	[233,234]
Ranolazine	Ranolazine	Inhibits the late sodium current, reducing intracellular calcium overload	Treats chronic angina by improving myocardial relaxation and reducing symptoms	[235,236]

## 9. Conclusions

This review highlights the multifaceted nature of cardiovascular diseases (CVDs), encompassing a range of conditions from atherosclerosis and hypertension to ischemic heart disease, heart failure (HF), cardiac arrhythmias, and cerebrovascular diseases. Understanding these conditions’ diverse manifestations is crucial given their profound impact on global health. We discussed various cardiac biomarkers, including troponins, BNP, CRP, CK-MB, H-FABP, galectin-3, and sST2, which are critical for accurate diagnosis and monitoring. The exploration of advanced imaging techniques, such as CT, PET, SPECT, and MRI, demonstrates their significant role in precise diagnosis and effective treatment planning. Synthetic inhibitors, including ACE inhibitors, statins, antiplatelet agents, ARBs, and beta blockers, are fundamental to CVD management, although their use is associated with side effects like hypotension, gastrointestinal disturbances, and potential drug interactions. Complementing these approaches, the review emphasizes the potential of herbal remedies, including Digitalis, Allium, Hawthorn, Ginkgo, Centella, and Salvia, alongside compounds such as quercetin, resveratrol, epigallocatechin gallate, and allicin. These remedies offer promising cardiovascular protective effects and may serve as complementary options to conventional therapies. However, it is crucial to emphasize that herbal remedies are supplements and not official or recommended therapies for CVD. Patients should consult their physician before incorporating any herbal supplements into their treatment regimen to ensure safety and avoid potential interactions with prescribed medications.

In conclusion, the integration of advanced imaging, biomarkers, pharmacological agents, and herbal remedies represents a comprehensive strategy for managing CVDs. Future research should focus on refining diagnostic tools, optimizing treatment protocols, and exploring the synergistic potential of herbal remedies in conjunction with conventional therapies. Continued innovation and interdisciplinary collaboration are crucial for advancing our understanding and management of cardiovascular diseases, leading to improved patient outcomes and reduced global burden.

## Figures and Tables

**Table 1:** A summary of the key cardiovascular conditions along with their causes, symptoms, complications, and management options.

Cardiovascular Condition	Primary Causes	Key Symptoms	Potential Complications	Management Options	References
Coronary Artery Disease (CAD)	Atherosclerosis (plaque buildup in coronary arteries)	Chest pain (angina), shortness of breath, fatigue	Myocardial infarction (heart attack), heart failure (HF), arrhythmias	Lifestyle changes, medications (statins, beta blockers), angioplasty, coronary artery bypass grafting (CABG)	[11,12,13,14]
Peripheral Arterial Disease (PAD)	Atherosclerosis affecting arteries in the limbs, typically the legs	Leg pain while walking (claudication), numbness, weakness	Critical limb ischemia, non-healing wounds, gangrene, amputation, increased risk of heart attack and stroke	Lifestyle changes, medications (antiplatelets, statins), revascularization procedures (angioplasty, bypass surgery)	[15,16]
Deep Vein Thrombosis (DVT) and Pulmonary Embolism (PE)	Formation of blood clots in deep veins, typically in the legs; clot dislodgment causing blockage in pulmonary arteries	Pain, swelling, redness, warmth in the affected limb (DVT); sudden shortness of breath, chest pain, rapid heart rate (PE)	Post-thrombotic syndrome, pulmonary hypertension, right ventricular failure, death	Anticoagulants, thrombolytics, compression stockings, surgical interventions (in severe cases)	[17,18]
Cardiomyopathies	Genetic factors, viral infections, alcohol abuse, certain medications, and other underlying diseases (e.g., hypertension, diabetes)	Fatigue, shortness of breath, swelling of the legs and ankles, palpitations	Heart failure, arrhythmias, sudden cardiac death	Medications (beta blockers, ACE inhibitors), lifestyle changes, devices (pacemaker, ICD), heart transplantation in severe cases	[19,20]
Cerebrovascular Diseases (Stroke)	Ischemic stroke: blockage of blood vessels in the brain; hemorrhagic stroke: rupture of blood vessels in the brain	Sudden weakness or numbness, confusion, difficulty speaking, vision problems, severe headache	Long-term physical and cognitive impairments, emotional disturbances, recurrent strokes	Thrombolytic therapy (ischemic stroke), surgical interventions (hemorrhagic stroke), rehabilitation and lifestyle modifications	[21]
Rheumatic Heart Disease (RHD)	Chronic valve damage following acute rheumatic fever, often caused by streptococcal infections in childhood	Shortness of breath, chest pain, fatigue, swelling in legs and abdomen	Atrial fibrillation, infective endocarditis, heart failure, stroke	Long-term antibiotics, medications for heart failure, valve repair or replacement surgery	[22,23]
Congenital Heart Defects (CHDs)	Genetic factors, maternal health conditions, and environmental exposures during pregnancy	Cyanosis, heart murmurs, shortness of breath, poor growth and development in children	Heart failure, arrhythmias, impaired growth and development, respiratory infections	Surgical repair or reconstruction, catheter-based interventions, long-term monitoring and medical care	[24,25,26,27,28,29]
Myocardial Infarction (MI)	Blockage of blood flow to the heart muscle due to a blood clot, often from a ruptured atherosclerotic plaque in a coronary artery	Severe chest pain, shortness of breath, sweating, nausea, light-headedness	Heart failure, arrhythmias, increased risk of recurrent MI, impaired quality of life	Immediate revascularization (angioplasty, CABG), thrombolytics, antiplatelet and anticoagulant therapy, lifestyle modifications	[30,31,32,33]
Cardiac Arrhythmias	Structural heart changes, electrolyte imbalances, ischemic heart disease, genetic predispositions	Palpitations, dizziness, fatigue, syncope, chest pain	Stroke (especially in atrial fibrillation), sudden cardiac arrest, heart failure	Medications (antiarrhythmics, anticoagulants), catheter ablation, pacemaker or ICD implantation, lifestyle changes	[34,35,36,37]

**Table 2:** Cardiac biomarkers and their primary roles and clinical applications in cardiovascular diagnosis/prognosis.

Biomarker	Type	Primary Role/Function	Diagnostic/Prognostic Utility	Associated Conditions	References
Cardiac Troponins (cTnI, cTnT)	Protein	Detects myocardial injury, diagnosis of AMI, and monitoring of chronic heart disease	Gold standard for diagnosing MI; elevated levels indicate cardiac muscle damage	AMI, chronic heart conditions, and HF	[38,39,40]
CK-MB	Enzyme	Detection of reinfarction; differentiation of new vs. ongoing cardiac injury	Useful for detecting reinfarction due to rapid rise and fall; less specific than troponins due to elevation in skeletal muscle injury	AMI, skeletal muscle damage	[41,42,43]
B-Type Natriuretic Peptide (BNP)/NT-proBNP	Hormone	Differentiation of heart failure (HF) from other causes of dyspnea; monitoring HF severity and treatment	Elevated levels correlate with HF severity; used for diagnosis, prognosis, and monitoring response to therapy, cardiac stress, and ventricular dysfunction	HF, cardiac stress, and ventricular dysfunction	[44,45,46,47,48,49,50]
Mid-Regional Pro-Atrial Natriuretic Peptide (MR-proANP)	Protein fragment	Diagnosis and risk stratification of HF; assessment of cardiac wall stress and fluid overload	Useful in differentiating HF from other dyspnea causes elevated levels associated with higher risk of hospitalization and mortality	Heart failure	[51,52,53]
Mid-Regional Pro-Adrenomedullin (MR-proADM)	Protein fragment	Assessment of cardiovascular health, vascular dysfunction, and systemic inflammation	Elevated in response to cardiovascular stress and inflammation. Useful in critical care for early identification of complications, such as sepsis or acute HF	Heart failure, sepsis, systemic inflammation	[54,55,56,57]
C-Reactive Protein (CRP)/hs-CRP	Protein	General marker of inflammation; assessment of cardiovascular risk.	Useful in assessing the inflammatory component of atherosclerosis. High hs-CRP levels predict future cardiovascular events independent of cholesterol levels	Atherosclerosis, acute coronary syndrome (ACS), HF	[58,59,60]
Heart-Type Fatty Acid-Binding Protein (H-FABP)	Protein	Reflects myocardial injury and oxidative stress	Early marker for myocardial infarction; assessment of myocardial injury; useful in the early detection of acute coronary syndromes	AMI, ACS	[61,62,63]
Myeloperoxidase (MPO)	Enzyme	Assessment of inflammation and oxidative stress in cardiovascular diseases, risk stratification in ACS	Elevated MPO levels are linked to plaque instability, endothelial dysfunction, and increased risk of cardiovascular events	ACS, atherosclerosis	[64,65,66,67]
Myoglobin	Protein	Early diagnosis of MI	Early release into the bloodstream aids in prompt MI diagnosis, monitoring muscle injury	MI, skeletal muscle injury	[68,69,70]
Ischemia-Modified Albumin (IMA)	Protein	Signals myocardial ischemia	Indicates early ischemic changes before myocardial necrosis; useful for early detection of ACS	ACS, myocardial ischemia	[71,72,73]
Galectin-3	Glycoprotein	Involved in inflammation and fibrosis	High levels indicate worse outcomes in HF; aids in assessing disease severity, prognosis, and monitoring response to therapy	Heart failure (HF), cardiac remodeling	[74,75,76,77]
Copeptin	Peptide	Reflects neurohormonal activation	Provides early diagnostic and prognostic information in ACS and HF; rises rapidly following myocardial injury; useful for early MI detection	Acute myocardial infarction (AMI), heart failure (HF)	[78,79,80]
Fibroblast Growth Factor 23 (FGF-23)	Hormone	Regulates mineral metabolism and is linked to cardiovascular pathology	Elevated levels predict adverse cardiovascular outcomes, especially in CKD and HF; associated with LV hypertrophy and vascular calcification	Chronic kidney disease (CKD), heart failure (HF)	[81,82,83]
Growth Differentiation Factor-15 (GDF-15)	Cytokine	Reflects cellular aging and systemic inflammation	Elevated levels are linked to increased cardiovascular risk; provides prognostic information in HF, CAD, and other cardiovascular conditions	HF, CAD, atrial fibrillation	[84,85,86]
Soluble ST2 (sST2)	IL-1 receptor	Indicator of cardiac stress and remodeling	Elevated levels correlate with worse HF outcomes; valuable for risk stratification and monitoring treatment efficacy	HF, cardiac remodeling	[87,88]
Endothelin-1 (ET-1)	Peptide	Potent vasoconstrictor involved in regulating vascular tone and blood pressure	Elevated levels are associated with hypertension, HF, and atherosclerosis; contributes to endothelial dysfunction and vascular remodeling	Hypertension, HF, atherosclerosis	[89,90]
Cardiac Myosin-Binding Protein-C (cMyBP-C)	Protein	Involved in sarcomere function in cardiomyocytes	Emerging biomarker for early diagnosis of myocardial infarction; elevated levels correlate with cardiac injury	Acute myocardial infarction (AMI)	[91]
Trimethylamine-N-oxide (TMAO)	Amine oxide	Metabolite produced by gut microbiota from dietary choline and carnitine	High levels are associated with an increased risk of cardiovascular events, including atherosclerosis and heart failure	Atherosclerosis, HF	[92]
Adiponectin	Hormone	Anti-inflammatory adipokine; regulates glucose levels and fatty acid breakdown	Low levels are associated with obesity, insulin resistance, and increased cardiovascular risk; higher levels are cardioprotective	Metabolic syndrome, vascular health	[93,94]
Interleukin-6	Cytokine	Pro-inflammatory cytokine	High levels are associated with increased cardiovascular risk and adverse outcomes in HF; marker of systemic inflammation	HF, atherosclerosis	[95,96]
TNF-α	Cytokine	Key cytokine in systemic inflammation	Elevated levels reflect inflammatory processes in HF and atherosclerosis; potential targets for anti-inflammatory therapies	HF, atherosclerosis	[97,98]
MicroRNAs (miRNAs)	Nucleotides	Involved in gene regulation during cardiovascular diseases	Specific miRNAs serve as novel biomarkers for the diagnosis and prognosis of AMI, arrhythmia, and HF; provide insights into molecular mechanisms	AMI, arrhythmia, and HF	[99,100,101]
Homocysteine	Amino acid	Amino acid linked to endothelial dysfunction	Elevated levels are associated with increased cardiovascular risk; contributes to atherosclerosis and thrombus formation	CAD, stroke, PAD	[102]
Phospholipase A2 (PLA2)	Enzyme	Enzyme involved in inflammation and atherosclerosis	High levels contribute to plaque formation and instability; associated with increased cardiovascular risk and plaque rupture	Atherosclerosis, CAD	[103]
Oxidized Low-Density Lipoprotein (oxLDL)	Lipoprotein	Marker of oxidative stress and atherosclerosis	Promotes inflammation and plaque formation; high levels indicate increased risk of atherosclerosis and CVD	Atherosclerosis	[104]
Lipoprotein (a) [Lp(a)]	Lipoprotein	Variant of LDL cholesterol; highly atherogenic	Elevated Lp(a) levels are an independent risk factor for atherosclerosis, myocardial infarction, and stroke	Atherosclerosis, MI, stroke	[105]
Apolipoproteins (ApoA1, ApoB)	Lipoprotein	Key components of lipoprotein particles; ApoA1 associated with HDL, ApoB with LDL	ApoB/ApoA1 ratio provides a better risk assessment of cardiovascular events than traditional lipid measurements	Atherosclerosis, MI	[106]
Oxylipins	Lipid mediator	Bioactive lipid mediators valuable biomarkers for detecting and monitoring cardiovascular diseases	Play a role in inflammation, vascular function, platelet aggregation, and leukocyte adhesion	Atherosclerosis, hypertension	[107,108]

**Table 3:** The key aspects of various cardiac imaging modalities, including their principles, applications, advantages, and disadvantages.

Imaging Modality	Principle	Applications	Advantages	Disadvantages	References
Chest X-ray	X-rays	Detect heart enlargement Identify fluid in lungs Diagnose other chest abnormalities	Quick and accessible Cost effective Provides an overview	Limited sensitivity Lacks detailed information about heart structures and function	[109,110]
Electrocardiogram (ECG)	Electrical activity of the heart	Evaluate heart rate and rhythm Monitor cardiac conditions Diagnose arrhythmias Detect myocardial infarction and identify electrolyte imbalances Assess pacemaker function	Inexpensive Widely available Non-invasive Essential for initial cardiac assessment and monitoring	Limited structural information May require complementary diagnostic tools	[111,112,113,114,115,116,117,118]
Echocardiography/ Ultrasound	Ultrasound waves	Diagnose heart valve diseases Monitor cardiomyopathies Detect congenital heart defects Assess ischemic heart disease Stress echocardiography 3D echocardiography Contrast-enhanced echocardiography	Non-invasive Real-time imaging No radiation Detailed assessment of heart structure and function	Image quality affected by body habitus and lung interference	[119,120,121,122,123,124,125,126,127,128]
Computed Tomography (CT)	X-rays	Assess congenital heart defects Characterize cardiac tumors Detect aortic aneurysms Coronary artery disease (CTA) Coronary calcium scan CT pulmonary angiography —CT FFR	High-resolution images Quick and suitable for emergencies Comprehensive anatomical evaluation	Exposure to ionizing radiation The use of contrast agents may be problematic for certain patients	[129,130,131,132,133,134,135,136,137]
Positron Emission Tomography (PET)	Radioactive tracers	Evaluate myocardial perfusion Distinguish viable vs. non-viable heart tissue Detect inflammation and infection Hybrid imaging (PET/CT, PET/MRI) Absolute quantification of myocardial blood flow	High sensitivity and specificity Detailed functional information Combines structure and function assessment	Radiation exposure Expensive and limited availability Time consuming and requires specific preparation	[138,139,140,141,142]
Single-Photon Emission Computed Tomography (SPECT)	Gamma rays	Assess myocardial blood flow Diagnose coronary artery disease Viability assessment post-myocardial infarction Evaluate left ventricular function Gated SPECT SPECT/CT hybrid imaging	Provides crucial insights into myocardial blood flow and function Combines perfusion and structural information	Lower spatial resolution compared to PET Time consuming and involves radioactive tracers	[143,144,145,146,147,148]
Magnetic Resonance Imaging (MRI)	Magnetic fields and radio waves	Diagnose cardiomyopathies Assess congenital heart defects Identify myocardial infarction and scar tissue Detect myocarditis and pericarditis Evaluate valvular diseases Stress cardiac MRI T1 Mapping Late Gadolinium Enhancement (LGE)	High-resolution images No ionizing radiation Comprehensive functional and anatomical evaluation	Expensive Limited availability Lengthy procedure Contraindications for certain implants and severe claustrophobia	[149,150,151,152,153,154,155,156,157]

## Data Availability

The data presented in this study are available upon request from the corresponding author. The data are not publicly available due to privacy restrictions.
